# The Multiple Functions of Rho GTPases in Fission Yeasts

**DOI:** 10.3390/cells10061422

**Published:** 2021-06-07

**Authors:** Jero Vicente-Soler, Teresa Soto, Alejandro Franco, José Cansado, Marisa Madrid

**Affiliations:** Yeast Physiology Group, Departamento de Genética y Microbiología, Facultad de Biología, Universidad de Murcia, 30100 Murcia, Spain; jerovic@um.es (J.V.-S.); teresaso@um.es (T.S.); afranco@um.es (A.F.)

**Keywords:** fission yeasts, Rho GTPases, guanine–nucleotide exchange factor (GEF), GTPase-activating protein (GAP), Cdc42, Rho1, cytoskeleton, polarity, cellular integrity, cytokinesis, sexual differentiation, crosstalk, signaling, phosphorylation

## Abstract

The Rho family of GTPases represents highly conserved molecular switches involved in a plethora of physiological processes. Fission yeast *Schizosaccharomyces pombe* has become a fundamental model organism to study the functions of Rho GTPases over the past few decades. In recent years, another fission yeast species, *Schizosaccharomyces japonicus*, has come into focus offering insight into evolutionary changes within the genus. Both fission yeasts contain only six Rho-type GTPases that are spatiotemporally controlled by multiple guanine–nucleotide exchange factors (GEFs) and GTPase-activating proteins (GAPs), and whose intricate regulation in response to external cues is starting to be uncovered. In the present review, we will outline and discuss the current knowledge and recent advances on how the fission yeasts Rho family GTPases regulate essential physiological processes such as morphogenesis and polarity, cellular integrity, cytokinesis and cellular differentiation.

## 1. Introduction

Rho GTPases are highly conserved proteins that regulate the actin cytoskeleton organization and morphogenesis in all eukaryotes and play central roles in many other physiological processes [1,2]. The fission yeast *Schizosaccharomyces pombe* has been widely used for deciphering the multiple cellular functions of the Rho GTPases, which are evolutionary conserved. *S. pombe* genome codes for six Rho GTPases, named Rho1 to 5 and Cdc42, that show 59 to 91% of identity at their amino acid sequences. The *Saccharomyces cerevisiae* counterparts, Rho1 and Cdc42, are essential [3,4]. They coordinately regulate cell wall biosynthesis and actin organization to guarantee cell integrity during vegetative growth [5]. Rho GTPases are equally crucial during cytokinesis, promoting ring constriction, septum formation and dissolution (recently reviewed in [6]). Sexual differentiation, in which cells generate a tip projection required for cell fusion, also requires the function of Rho GTPases [7].

*Schizosaccharomyces japonicus* is another fission yeast species that has become a model organism for exploring developmental and physiological evolutionary changes within the genus *Schizosaccharomyces* [8]. Although both *S. pombe* and *S. japonicus* share a binary fission mode of cell division, *S. japonicus* possesses several interesting and distinctive features [9]. One of the most relevant is that it can transit from unicellular yeast to filamentous hyphae and form a true mycelium [10], thus making this genetically tractable non-pathogenic yeast a model for investigating the requirements of invasive hyphal growth. While most of our current knowledge on Rho GTPases functions in fission yeast has been obtained from studies with *S. pombe*, recent works on *S. japonicus* have unveiled how the mechanisms controlling growth are reorganized to produce different cell shapes and structures [11,12].

In this review, we present the current status and recent advances in the plethora of functions in which these molecular switches are involved in both fission yeasts species, and the mechanisms of functional crosstalk that ensure signal specificity, coordination, and fidelity of the different controlled cellular processes.

## 2. Rho GTPase Regulation

Rho GTPases act as molecular switches cycling between an inactive GDP-bound and an active GTP-bound conformation in response to physical and chemical stimuli. In the GTP-bound active form, Rho GTPases associate with the plasma membrane and selectively interact with a range of effectors, including kinases, actin regulators and many others, leading to changes in cell behavior. Cycling is regulated by guanine nucleotide exchange factors (GEFs) that catalyze GDP exchange for GTP, thereby activating the GTPase. In contrast, GTPase-activating proteins (GAPs) accelerate the intrinsically slow hydrolysis of GTP, causing its inactivation [13]. Guanine nucleotide dissociation inhibitors (GDIs) sequester the GDP-bound form of some GTPases in the cytosol to avoid their activation by GEFs or localization to membranes [13].

The number of different GEFs and GAPs outnumbers the Rho GTPases in most organisms, including fission yeasts [14]. Therefore, several Rho regulators can impart specialized functions to individual Rho GTPases. GEFs and GAPs can also coexist in regulatory complexes to fine-tune and maintain optimal levels of Rho GTPase signaling.

Besides the classical GTP-GDP cycling, Rho GTPases are regulated by multiple factors that contribute to the complexity of Rho GTPase signaling, such as the crosstalk between its family members, the subcellular distribution of GEFs and GAPs, changes in gene expression or post-transcriptional regulation (reviewed in [15,16]), and post-translational modifications, including lipid modifications, phosphorylation, sumoylation, and ubiquitylation, that regulate the Rho GTPases stability and spatial distribution (see [1] for an excellent review). These multiple layers of regulation are orchestrated in response to different signals, providing a specific and precise signature of Rho GTPase activation that will depend on the physiological context of the cell.

## 3. Rho GTPases and Their Regulators in *Schizosaccharomyces pombe*

**Figure 1:**
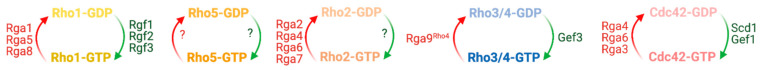
*S. pombe* Rho GTPases and their regulators. See main text and tables for details on each protein.

*S. pombe* has six Rho GTPases (summarized in Figure 1, Table 1). The essential GTPases Cdc42 and Rho1 have been extensively studied, with Cdc42 playing a fundamental role in establishing cell polarity and morphology, whereas Rho1 is critical for cell wall synthesis and septum formation, to which Rho2 also contributes. Rho3 and Rho4 regulate secretion and exocytosis, while Rho5 is a paralogue of Rho1 that shares similar functions.

### 3.1. Rho1

*S. pombe* Rho1 is essential for viability and is a functional homolog of human RhoA and budding yeast Rho1 [98]. It participates in the coordination of actin polarization with the cell wall biosynthesis. The best-characterized effectors of Rho1 are the β(1,3)-glucan synthase (GS) complex [3] and the PKC orthologs Pck1 and Pck2 (Section 5) [18,20]. Rho1, through Pck1, also cooperates with Rho2-Pck2 in the activation of the Cell Integrity MAP kinase pathway (CIP) (Section 5) [21]. Rho1 activity is a critical regulator of the actin cytoskeleton, although no specific targets have been described yet [17,98]. This GTPase is activated by three GEFs named Rgf1, Rgf2 and Rgf3 (see Table 2 for more details) [19,25,26,27], which are structural orthologs of budding yeast Rho1-GEFs Rom1 and Rom2 [99,100]. Another GEF named Gef2 is also important during cytokinesis and binds Rho1, Rho4 and Rho5 in vitro [101,102]. However, its putative role as a Rho1 activator in vivo is unknown (Table 2). The downregulation of Rho1 activity relies on three GAPS: Rga1, Rga5 and Rga8 (Table 3). None of them are essential for cell viability [103].

### 3.2. Rho2

Rho2 GTPase is 53.2% identical to RhoA and budding yeast Rho2, whereas Rho1 and Rho2 share 52.3% identity [104]. Rho2 controls cell polarity, actin cytoskeleton organization, and cell wall biosynthesis (Table 1) [32,35,37]. It activates the α-(1,3)-GS Mok1 via the protein kinase C (PKC) ortholog Pck2 (Section 5) [32,35,37]. Moreover, Rho2 and Pck2 are the primary positive regulators operating upstream of the CIP (Section 5) [34,38]. Rho2 is negatively regulated by four GAPs (Rga2, Rga4, Rga6 and Rga7), all of which also downregulate the CIP (see Table 3) [105]. No Rho2 GEFs have been described so far in *S. pombe*.

### 3.3. Rho3

Rho3 GTPase plays essential roles during *S. pombe* polarized growth and exocytosis (see Table 1) [40,41,42], and has also been involved in the regulation of Golgi/endosome trafficking (Section 4) [45] and in sexual differentiation (Section 7) [46]. Gef3 is the only known Rho3 GEF and plays a role during cytokinesis [45], without promoting GTPase nucleotide exchange [45].

### 3.4. Rho4

Rho4 is involved in cell morphology, septation and cell wall integrity [47,48,49]. It regulates the polarized secretion of the glucanases Eng1 and Agn1 during cell separation in a non-redundant manner with Rho3 (Section 6) [48,49], and may be required for localization of the exocyst at the division area [50]. A role for Rho4 in the regulation of the actin cytoskeleton and cytoplasmic microtubules at 37 °C has also been described [47,48]. Rho4 also acts upstream of the CIP during vegetative growth (Section 5) [51]. The only GEFs known to interact with Rho4 are Gef2, Gef3 and Scd1 (Table 2) [102,106,107]. Rho4 also interacts with Rdi1, the sole Rho GDI in *S. pombe*, but Rdi1 is not essential for Rho4 localization [47]. Rho GAP Rga9 interacts by two-hybrid with Rho4 [47], and may cooperate with Rdi1 to negatively control Rho4 subcellular localization (Table 3) [47].

### 3.5. Rho5

Rho5 is a functional paralogue of Rho1 (86% amino acids identity), and likely functions redundantly with Rho1 during cell growth and division (Table 1) [52,53]. It is strongly induced during the stationary phase, and participates in spore cell wall formation during sporulation (Section 7) [53]. Moreover, Rho5 stimulates Pmk1 activation during vegetative growth (Section 5) [51]. The identity of Rho5 regulators and/or effectors is currently unknown.

**Table 2:** Guanine nucleotide exchange factors (GEFs) for Rho GTPases in fission yeast.

GEF	GTPase	Localization	Regulated/Involved Processes
Scd1	Rho4 Cdc42	Nucleus and mitotic spindle [108] and active growing sites: on the membrane at cell poles (interphase), division area (cytokinesis) [57,72,80,109,110]	**Morphology and cell polarity** [57,65]: oscillates between the two cell poles [67] and restricts Gef1 localization to sites of polarization to prevent ectopic Cdc42 activation (cytokinesis) and to maintain cell shape (interphase) [74] Involved in **endocytic trafficking** with Nrf1 [111] **Mitosis**: Involved in spindle formation with Moe1 [108,112] **Cytokinesis**: localizes later than Gef1 to the ingressing furrow and promotes septum formation [86] **Sexual differentiation**: mating [57,93]
Gef1	Cdc42	Active growing sites: cell poles (cytosol, active during interphase) and division area (cytokinesis) [75,80,113]	**Cell polarity**: enables NETO [59,67] promoting Scd1 recruitment at the new end to allow the transition from monopolar to bipolar growth [74] **Cytokinesis**: localizes before Scd1 to the actomyosin ring and promotes timely constriction [86], interacts with Hob3, which promotes cytokinesis [80] and promotes Scd1 localization to the division site through recruitment of Scd2 [74]
Gef2	Rho1 Rho4	Contractile ring Precursor cortical nodes [102,114]	**Cytokinesis**: involved in division-site and contractile-ring positioning by interacting with Mid1 [101] and contributes to the positioning of the division-site and contractile-ring stability together with Nod1 [102]
Gef3	Rho3 Rho4	Septin ring [45]	**Cytokinesis**: possible scaffold for septin-mediated Rho3-directed polarized secretion [45] and interacts with the septin complex and Mid2 and activates Rho4 [107]
Rgf1	Rho1 Rho2?	Active growing sites: cell poles and division area [19,27] Fully formed contractile rings [31] Cell nucleus during stalled replication by HU [115]	**Cell polarity**: regulates Rho1-mediated cell wall deposition during polarized growth [19] **Cell wall integrity**: activates CIP, via Rho1 and Pck2 [24] **Cytokinesis**: promotes Rho1 activation during septum formation [19,27], and participates in a cytokinesis checkpoint during cell wall damage, via Rho1 and Pck2-Pmk1 [31] **Other functions**: Its nuclear accumulation promotes tolerance and survival during replication stress [115], and is required for double-strand break repair via Rho1 [116]
Rgf2	Rho1	Periphery of the spore after meiosis I and II [117] Cell tips and septum during vegetative growth (upon mild overexpression) [26,27,117]	**Cell polarity**: secondary function (redundant to Rgf1): regulates Rho1-mediated cell wall deposition during polarized growth at least through Bgs2, but not exclusively [117] **Cytokinesis**: Rho1 activation during septum formation [25,26,27] **Sexual differentiation**: main function during assembly of the spore cell wall by activation of GS subunit Bgs2 [117]
Rgf3	Rho1	Ring/membrane interphase [27,114,118], depends on Art1 [119]	**Cell wall integrity**: essential for maintaining cell integrity during cell separation [25,26] **Cytokinesis**: activates Rho1 specifically during cytokinesis [25,26,27]. Regulated by transcription factor Ace2, which promotes its maximal expression during septation [25,26,120], and arrestin Art1 [119]

**Table 3:** GTPase activating proteins (GAPs) for Rho GTPases in fission yeast.

GAP	GTPase	Localization	Regulated/Involved Processes
Rga1	Rho1	Cell poles (interphase) and the division site (cytokinesis) [103]	Negative regulator of Rho1 and is involved in actin-patch localization, cell **morphogenesis**, **septation**, and **cell wall synthesis** [103]
Rga2	Rho2 Rho1 ^(+)^	Cell poles (interphase) and the division site at the ring/membrane interphase (cytokinesis). Localization depends on polarity markers and actin polymerization [36,114]	**Cell polarity and morphogenesis**: Positive effect, direct or indirect, in Cdc42 activation playing an antagonistic role to Rga4 to maintain cell dimensions [36] **Cell wall integrity**: Rga2 negatively regulates Rho2-Pck2 interaction and Pck2 stability. Rga2 acts as a negative regulator of the Rho2-Pck2 interaction with the CIP. Lack of Rga2 suppress the lysis of *mok1-664* at 32 °C [36]
Rga3	Rho1 ^(+)^ Cdc42	Cell poles and the division site with Cdc42-GTP during mitotic growth [73]. During sexual differentiation, Cdc42 patches and sites of polarity [73]	**Cell polarity**: paralogue of Rga4 and synergizes with Rga4 and Rga6 to restrict Cdc42-GTP zone sizes during mitotic growth [73] **Sexual differentiation**: limits the lifetime of unstable Cdc42-GFP patches important for the wandering motion that favors mating and confers a competitive advantage during sexual reproduction [73]
Rga4	Rho2 Cdc42	To the plasma membrane at the cell sides (interphase) and the division site (at the end of cytokinesis). Forms clusters at cell sides and nongrowing cell pole [121,122]	**Cell morphogenesis and polarity**: restricts Cdc42 activation at the cell sides controlling cell diameter and symmetry breaking [121,122]. Pom1 regulates its localization and phosphorylation [105,122]. Its exclusion from cell poles to allow bipolar Cdc42 activation also depends on Dis2 [123] **Cell wall integrity**: Rho2 GAP negatively regulates the activity of the CIP, which is not involved in the Rga4-dependent control of cell shape [105]. Not involved in Pom1 negative regulation of the CIP. Rga4 positively regulates cell wall integrity and cell separation independently of the Pmk1 pathway, acting as a Cdc42 GAP [105]
Rga5	Rho1	Cell poles (interphase) and the division site (cytokinesis) upon mild overexpression [124]	**Cell morphology**: participates in the regulation of cell morphology and cell wall biosynthesis at high temperature [124] **Cell wall integrity**: Specific Rho1 GAP that negatively modulates the Rho1-Pck1, and to a lesser extent Pck2 interaction, decreasing their stability. Negative regulation of 1,3-β-GS activity [124] **Cytokinesis**: cell separation defect and/or delay [124] **Sporulation**: *rga1*∆ *rga5*∆ spores are unable to germinate [124]
Rga6	Rho2 Cdc42	Plasma membrane at the cell sides, forming clusters different from those made by Rga4 and growing cell poles [125]	**Cell morphology and polarity**: Cdc42 GAP and collaborates with Rga4, although its role could be higher than Rga4 in the negative regulation of Cdc42 at the growing cell pole [125] **Cell wall integrity**: Rho2 GAP [36,105]
Rga7	Rho2	Cell poles (interphase) and the division site (cytokinesis) [126]	**Cell wall integrity**: Rho2 GAP involved in the negative regulation of the CIP [105] **Cytokinesis**: The GAP activity is dispensable for Rga7 function in cytokinesis [126]. Cooperates with Cdc15 and Imp2 in actomyosin ring stability and proper disassembly, and successful septum formation and separation to ensure cell integrity [126]. Participates with Rng10 in Bgs4 trafficking from the Golgi to plasma membrane adjacent to the contractile ring [127]
Rga8	Rho1	Cell poles (interphase) and the division site (cytokinesis) [128], with a monopolar pattern when Pak1 activity is abolished [128]	**Polarity**: Rho1 GAP and is a downstream target of Pak1 [128], and participates in the crosstalk between Rho1 and Cdc42 [5,128] Overexpression causes **morphological defects**, a **cytokinesis delay** and **cell lysis** (like *rho1^+^* overexpression) [128]
Rga9	Rho4 ^(+)^ Cdc42 ^(+)^		Rho4 GAP and may function cooperatively with Rdi1 to negatively control the cellular localization of Rho4 [47] and Cdc42 (GAP) [47]

^(+)^ in vitro binding partner.

### 3.6. Cdc42

The Cdc42 GTPase is involved in many aspects of growth and cell-cycle regulation, including actin cytoskeletal rearrangements and activation of signal transduction pathways (Section 4, Section 6 and Section 7) [78]. In fission yeast, lack of Cdc42 essential function results in arrested small and round uninucleate cells with mislocalized actin that cannot mate [4,129]. Unlike budding yeast, expression of constitutively active *cdc42* alleles in *S. pombe* is not lethal, but it results in an abnormal morphological phenotype of large, round or misshapen cells with delocalized cortical actin structures (Table 1) [4,130]. Cdc42 is activated by two GEFs with distinctive functions, Scd1 [57,131] and Gef1 [59,132], which are together essential for viability (see Table 2 for details). On the other hand, the GAPs Rga4, Rga6 and Rga3 catalyze GTP hydrolysis for Cdc42 inactivation (Table 3) [73,122,125]. Rga3 co-localizes with active Cdc42 at cell tips, whereas Rga4 and Rga6 are present at the cell sides. Despite its different localization, Rga3 synergizes with Rga4 and Rga6 to restrict the cellular localization of active Cdc42 during growth [73].

## 4. On the Role of Rho GTPases during Polarized Growth

Cell polarization results in the asymmetric organization of the cytoskeleton in response to internal and/or external cues. Polarized growth requires cell surface expansion, which results from multiple connected biochemical and biomechanical elements. They include surface material synthesis by exocytosis and endocytosis, plus mechanical components that set the elasticity of the cell surface, such as the actin cortex or the cell wall [133].

Fission yeast cells are cylindrical, grow by cell tip extension, and divide by medial fission. In these cells, polarized growth and cell wall synthesis are first restricted to the old cell tip after division, and cells grow in a unipolar manner until they reach a length of ~9.0–9.5 µm. Then, they initiate growth at the new end created during cytokinesis, a process named NETO (New End Take Off), establishing bipolar growth [72]. Thus, a new functional cell polarity area at the new tip is formed in the presence of a pre-existing one in the old tip. As to why polarized growth starts first in the old tip, the answer is not simple and is a fertile area of research. The fact that NETO can be delayed in several cytokinetic mutants suggests that cytokinetic remnants suppress initial polarization at the new end [134].

### 4.1. Schizosaccharomyces pombe

Cdc42 GTPase is the core upstream polarizing cue in fission yeast in most physiological contexts. However, a Cdc42-independent microtubule-mediated pathway that depends upon the polarity factor Tea1 mediates de novo polarization in fission yeast cells after starvation by determining the position of sterol-rich domains (SRMs) at the plasma membrane [135].

**Figure 2:**
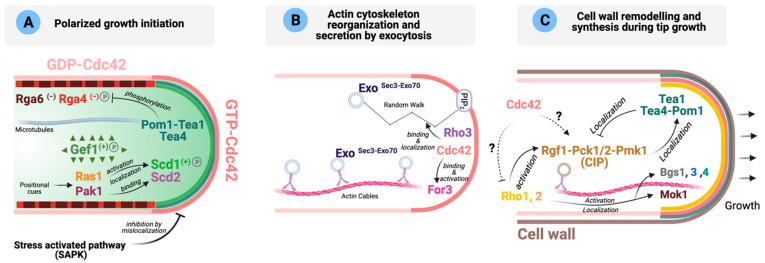
Role of Rho GTPases during *S. pombe* polarized growth (adapted from [77,146,147]). (**A**) Schematic representation of the main molecular players involved in the local activation of Cdc42 (GTP-Cdc42, dark pink) during polarized growth initiation. GAPs are shown in different shades of red, and GEFs in different shades of green. (−): negative regulation on Cdc42; (+): positive regulation on Cdc42; P: protein is phosphorylated. Please see text for details. (**B**) Activated Cdc42 binds and activates formin For3 promoting actin cytoskeleton reorganization. Exocytic vesicles can reach the cell tips either transported along actin cables by myosin V Myo52 or by random walk. Once there, Sec3 and Exo70 tether the exocyst and the vesicle by binding PIP_2_, Cdc42 and Rho3. Exo represents Sec5, Sec6, Sec8, Sec10, Sec15 and Exo84. (**C**) Cell wall remodeling is initiated after secretory vesicles containing the enzymes necessary for cell wall remodeling reach the poles. These enzymes are activated by GTP-Rho1 and -Rho2. The Cell Integrity MAPK Pathway (CIP) is involved in the regulation of cell wall homeostasis, and the polarity landmarks Tea1, Tea4 and Pom1 functionally interact. Dotted arrows mean that the molecular links have not been established. Please see main text for more details.

**Cdc42 activation and polarized growth initiation***. S. pombe* prenylated Cdc42 localizes ubiquitously at the plasma membrane, but it concentrates to the growing cell ends in a GTP-bound (active) form. Polar active Cdc42 is less mobile than the inactive GDP-bound isoform, which localizes primarily to the cell lateral sides (Table 1) (Figure 2A) [56,136,137]. Inhibition of Cdc42 activity at the lateral cortex is achieved by the negative regulation imposed by its GAPs Rga4 and Rga6, and is essential for polarity establishment since GEF-mediated local activation at the cell tips is not enough to restrict Cdc42 activity (Figure 2A) [67,138,139].

Two GEFs, Scd1 and Gef1, promote Cdc42 GTP-loading at the cell ends (Table 2, Figure 2A). Scd1 plays a critical role to control cell morphology by acting as the primary Cdc42 activator locally at the growing areas where it localizes. To this purpose, Scd1 forms part of a multiprotein complex composed of Ras1-Scd1-Scd2-Cdc42-Pak1 (see below) [57,110], which is similar to *S. cerevisiae* Bud1-Cdc24-Bem1-Cdc42-Ste20 complex. The other GEF, Gef1, is a cytosolic protein that associates with the membrane at the cell poles in response to certain stresses modulating Cdc42 activity under such conditions (Figure 2A). Gef1 is also important for the establishment of bipolar growth by promoting growth from the new cell end formed by the preceding cell division (NETO transition). In *gef1*∆ cells, both the Cdc42 GEF Scd1 and its scaffold Scd2 are localized mainly to the old ends, and the new pole cannot initiate polarized growth [59,74].

In fission yeast, active Cdc42 levels alternate over time between one pole and another in an anticorrelated manner (when active Cdc42 decreases at one end, it increases at the other) [67]. This oscillatory behavior has been attributed to the existence of GEF-mediated positive and GAP-dependent negative feedback mechanisms, and the anticorrelation indicates competition for active Cdc42 or its regulators [67]. Several studies support that positive feedbacks are important for the symmetric breaking required for polarization, especially when cells lack internal or external landmarks [71,93]. In *S. cerevisiae*, recent works have shown that positive feedback by local activation is necessary and sufficient for breaking its symmetry [140,141]. For instance, blocking local enrichment of Cdc24 (Scd1 ortholog) or Bem1 (Scd2 ortholog) by distributing them all over the membrane prevented Cdc42 polarization [141]. On the other hand, negative feedbacks are necessary for the Cdc42 dynamic oscillatory patterns observed during vegetative growth [67]. GAPs and the key downstream effectors PAK-family kinases have an important role in the negative-feedback regulation of Cdc42 in both *S. pombe* and *S. cerevisiae* [67,125,142,143,144].

As noted above, Cdc42 GEFs mediate the positive feedbacks required for polarization, in which fission yeast Scd1 plays a primary role. During interphase, active Cdc42 promotes positive feedback recruiting Scd1 through the scaffold Scd2. Recent data suggest that Gef1-mediated Cdc42 activation is required for Scd1-Scd2 recruitment to sites with no prior history of Cdc42 activation and growth (for instance, the new end) [74,76]. Scd2 promotes positive feedback enhancement of Cdc42 activity by binding Scd1, Pak1 and Cdc42-GTP (Figure 2A) [76]. Moreover, Scd1 is required to prevent ectopic Gef1 localization and loss of polarity [74]. Thus, Gef1 primes Cdc42 activation at new sites to initiate Scd1-dependent polarized growth, while Scd1 restricts Gef1 to areas of polarization [74]. This GEFs crosstalk could be a conserved mechanism that orchestrates precise Cdc42 activation during complex cellular processes [74]. Moreover, the small GTPase Ras1 participates in the activation of Cdc42 by providing a positional input through the recruitment and activation of Cdc42 GEF Scd1 (Figure 2A). Thus, Ras1 and Scd2-dependent positive feedback cooperate synergistically to yield robust zones of Cdc42 activity (Figure 2A) [76]. In mammalian cells, Ras also promotes GEF activity towards Rac, a Cdc42-related GTPase [145]. Hence, activation of Rac/Cdc42 GEFs might be an evolutionarily conserved function of Ras-family GTPases.

The localization of Scd1 and Gef1 is regulated post-translationally by phosphorylation. The NDR/LATS kinase Orb6 phosphorylates Gef1 and promotes its binding to 14-3-3 protein Rad24, which displaces Gef1 from the cortex, to reduce polarized activation of Cdc42 [61,70]. On the other hand, a recent phosphoproteomic study has identified Scd1 as one of the p21-activated serine/threonine kinase Pak1 targets involved in polarized growth [89]. Scd1 phosphorylation by Pak1 is important for the regulation of its nuclear shuttling [89]. In *S. cerevisiae*, it has already been described that the inhibitory phosphorylation of Cdc24 by PAKs is important in the negative feedback regulation of Cdc42 activity [143,148]. Future work will be needed to unravel the functional significance of Pak1-dependent Scd1 phosphorylation in fission yeast polarized growth.

As discussed earlier, the negative regulation of Cdc42 during polarized growth is mostly dependent on the GAPs Rga4 and Rga6. Both GAPs localize at the lateral cortex, where they are important for preventing active Cdc42-directed growth at cell sides, thereby restricting bipolar growth to the cell tips [121,122,123,125]. In *S. pombe*, local Cdc42 activation at the tips is defined by microtubules [122,123,149,150,151,152,153], whereas in *S. cerevisiae,* Cdc42 activation at specific sites depends on a set of locally deposited landmarks during the growth of the bud that remain there in the newly born cell [154]. Fission yeast microtubules transport and deposit the tip factors Tea1 and Tea4 at the cell poles [149,150,151,155]. Tea4 acts as a phosphatase regulatory subunit by recruiting the type I phosphatase (PP1) subunit Dis2 to the cell ends to promote the local dephosphorylation of DYRK-family kinase Pom1, so revealing a membrane-binding region [152,153,156]. Dephosphorylated Pom1 is associated with the plasma membrane at cell tips, where it is essential for the exclusion of the Cdc42 GAP Rga4 from the nongrowing cell end and bipolar Cdc42 activation [122]. Pom1 physically interacts with Rga4 and affects its phosphorylation state, although the exact molecular mechanism of Rga4 regulation by Pom1 remains to be elucidated [122]. The Tea4-PP1 complex might also favor local Cdc42 activation and polarized growth by excluding Rga4 and recruiting Gef1, probably by reversing Gef1 phosphorylation by Orb6 [61,70], independently of For3 and Pom1 (Figure 2A) [123].

Other kinases beyond Pom1 are also involved in modulating Rga4 localization. Recent data have shown that the Cdc42 effector Pak1, unlike Pom1, directly phosphorylates Rga4 [89]. In the absence of Pom1 and Pak1, Rga4 becomes enriched at both cell tips, indicating that both polarity kinases collaborate as tip exclusion factors and that cells require active repulsion of this GAP at cell ends to sustain polarized growth [89]. Orb6 might also be involved in Rga4 phosphorylation to modulate Cdc42 activation, thus controlling cell dimensions and growth symmetry [61,121]. More recently, it has been suggested that Rga4 might localize to the cell ends to block Cdc42 activation and growth during mitosis [157]. Taken together, all this evidence supports the idea that Rga4 plays an essential role in locally restricting Cdc42 activity.

The Cdc42 GAP Rga6 is also functionally important synergizing with Rga4 during the control of cell polarity. In the absence of both Rga6 and Rga4, cells are rounded and show active Cdc42 all around the membrane [125]. Rga6 localizes to the lateral cell cortex, forming clusters different from those made by Rga4, and reaches the growing tips where it might be regulated by degradation [125]. The observed reduced concentration of Rga6 at the growing tips is likely due to the polarized secretion of Scd1 mediated by formin For3 nucleated actin cables, which locally regulates the decrease of Rga6 [125]. At the tip, Rga6 modulates the amplitude of active Cdc42 oscillations, and might participate in the negative feedback regulation mediated by the Scd1-Scd2-Pak1 complex [67,125]. Lack of Rga6 increases the amount of old tip-bound Cdc42-GTP, and decreases the active Cdc42 symmetry required for bipolar growth. However, these alterations are not strong enough to elicit a severe monopolar growth, and cells only show a slightly wider diameter and decreased growth at the new end [125]. Therefore, both GAPs collaborate to spatially restrict active Cdc42 at the cell tips but with a predominant role of Rga6 in the negative regulation of Cdc42 at the growing cell tip. A recent study has identified Rga6 as an interacting protein of the septin complex that promotes Spn1 septin localization at the cell cortex, particularly to the region near the growing cell tip [158], and it has been proposed that the diffusion-barrier function of septins and Rga6 GAP activity might be integrated to regulate polarized localization of active Cdc42 [158]. In *S. cerevisiae*, recruitment of septins to the budding site depends on the Cdc42 effectors Gic1 and Gic2 [159]. Once there, septins recruit the GAP proteins to inactivate Cdc42 as an additional negative feedback mechanism [144]. However, in fission yeast, the absence of Spn1 does not appear to affect the localization of Rga6 [158]. Thus, Cdc42 GAP protein and septin interaction seems to be evolutionary conserved, although it occurs differently according to the organism. In higher eukaryotes, it is unknown whether Rho GAPs also regulate the cortical localization and function of septins. Another function of Rga6 in polarized growth might be to link the growth history of the mother cell to Cdc42 activation in daughter cells [160].

The interplay between Cdc42 GEFs and GAPs precisely controls Cdc42-mediated polarized growth. Both Scd1 and its scaffold Scd2 and the Cdc42 GAP Rga4 seem to act additively to define the dimensions of the growth zone, since the lack of both proteins gives rise to round cells [65]. Moreover, the respective levels of Gef1 and Rga4 proteins at the membrane define the growing area dynamically at each cell tip [65,66,70]. Recent optogenetic studies based on the CRY2-CIB system have revealed that in mammalian cells, Rac1 and RhoA become active at the cell cortex upon light-dependent cytosolic clustering [161]. In these experiments, the small GTPases were fused to CRY2PHR (simply denoted CRY), the photolyase homology region of *Arabidopsis thaliana* cryptochrome 2, which oligomerized upon blue light exposure [162]. Using a similar approach, it has been described that in fission yeast, CRY2-dependent clustering at the membrane promotes Cdc42 activation at lateral sites, where Cdc42 is usually inactive [163]. Although activated clustered Cdc42 can recruit Scd1 through the scaffold Scd2, the positive feedback does not become established because it is counteracted by Rga4 GAP-mediated inactivation. These results highlight the cell polarization system’s robustness and the importance of the interplay between GEFs and GAPs in establishing polarized growth [163].

**Cdc42 effectors and polarized growth**. Active Cdc42 coordinates polarized growth at cell tips by translating the polar localization of the actin cytoskeleton and the exocytosis machinery into actual growth [64]. Locally active Cdc42 targets the delivery of new plasma-membrane material and cell wall-remodeling enzymes through recruitment and activation of its effectors, the p21-activated kinase (PAK) Pak1, formin For3 for nucleation of actin cables, and the exocyst complex for polarized exocytosis (Figure 2A,B) [68].

The p21-activated serine/threonine kinases (PAKs) comprise a family of protein kinases that are highly conserved amongst eukaryotes. PAKs bind to the activated (GTP-bound) forms of Cdc42 and Rac, but not to other small GTPases such as Ras or Rho, and are activated as a result of this binding [164]. In fission yeast, there are two known PAK homologs, Pak1/Shk1/Orb2 [130,165,166] and Pak2/Shk2 [164,167]. Pak1 is a 72-kDa protein homolog of the *S. cerevisiae* Ste20 and mammalian Cdc42/Rac-binding kinase, p65^PAK^ protein kinases which mediates functions of the Ras/Cdc42 signaling complex [165]. Pak1 is essential for cell viability [130] and localizes to the cell ends and to the actomyosin ring at the cell division site [81,168]. The second protein, Pak2, is a structural and functional homolog of Pak1 which has the greatest similarity in predicted amino acid sequence to *S. cerevisiae* Cla4 and Skm1. Like Pak1, Pak2 interacts physically and functionally with Cdc42 and it is involved in Ras1-Cdc42-mediated morphological control and mating response pathways [164,167]. However, its lack does not cause a phenotypic change and its functions are partially redundant with those of Pak1, with Pak1 being functionally dominant [164,167].

Pak1 contains an N-terminal autoinhibitory regulatory domain and a C-terminal protein kinase catalytic domain [169,170]. Kinase activation follows interaction with the small GTPases Cdc42, which bind to a CRIB (Cdc42 and Rac interactive binding) motif within the regulatory domain [171]. The binding of Cdc42 disrupts the intramolecular interactions of Pak1, thereby removing the autoinhibitory effect [169]. The scaffold Scd2 facilitates the interaction of active Cdc42 with Pak1 [109]. At the same time, Pak1 regulates Cdc42 activity likely by altering the intracellular distribution of Scd2 and the Cdc42-GEF Scd1 within the multiprotein complex constituted by Ras1-Scd1-Scd2-Cdc42-Pak1 [67]. Other proteins involved in polarized growth are phosphorylated in a Pak1-dependent manner in cells, such as the microtubule end-associated factor Tea1 [172] and Tea3, a scaffold protein for cell polarity proteins at cell ends [173]. Tea3 is part of the Pak1-dependent negative feedback loop, and prevents pre-existing growth domains from becoming overpowering by competing with Scd2 for its binding to Pak1 [173]. In the absence of Tea3, GTP-Cdc42 oscillations are impaired and NETO is delayed [173]. Pak1 also phosphorylates the Rho1 GAP Rga8 [128], which points to a crosstalk between Rho1 and Cdc42.

Another important Cdc42 effector is For3, a non-essential diaphanous-like formin that assembles actin cables for cellular transport and has established roles in polarized secretion and growth during interphase (Figure 2B) [58,60,174,175]. The class V myosin Myo52 transports along with these cables vesicular cargoes containing enzymes necessary for cell wall remodeling and polarized growth at cell tips [58,176,177,178]. For3 localization to the growing ends depends on active Cdc42 and the actin nucleation promoting factor Bud6, and it occurs through a complex regulation that involves N-terminal and C-terminal domains [60,179]. As expected, hypomorph Cdc42 alleles show a severe defect in actin cables and For3 localization [60,62]. For3 neither localizes to the cell poles nor binds to its cortical tethers when found in an autoinhibited (closed) state, which is mediated by an intramolecular interaction between the autoregulatory (DAD) and inhibitory (DID) domains [60]. GTP-loaded Cdc42 relieves the autoinhibited state of For3 to an active conformation [60]. The GTPase Rho3 also interacts with For3 and contributes to regulate the actin cytoskeleton [40]. In *S. cerevisiae*, besides Cdc42, Rho1 and Rho3 participate in the assembly of actin cables mediated by the formins Bni1 and Bnr1 [180].

Additionally, For3 depends on the linker protein Tea4 for normal localization to both cell ends [149,150], which might be a crucial step for growth at the new end, as cells lacking this formin maintain a polarized growth but show altered morphology and a defect in NETO [149]. Accordingly, artificial targeting of For3 to the cell ends is sufficient to restore bipolar growth in the absence of Tea4 [149]. Moreover, *for3*∆ mutants show an altered growth pattern similar to that of cells lacking Rga4, the major Cdc42 GAP, which is required for normal For3 localization [121].

Cdc42 also plays a key role to control polarized exocytosis (Figure 2B). The exocyst, a pivotal eight-subunit tethering complex (composed by subunits Sec3, Sec5, Sec6, Sec8, Sec10, Sec15, Exo70 and Exo84), is conserved from yeasts to mammals, and is involved in the late stages of exocytosis by the targeting and tethering of post-Golgi secretory vesicles to the plasma membrane [181,182]. In fission yeast, localization of the exocyst to the cell tips depends on either myosin V-directed transport along For3-nucleated actin cables [146] or random walk (Figure 2B) [64], with both mechanisms playing complementary roles for polarized exocytosis. At the cell poles, exocyst subunits Sec3 and Exo70 tether the exocyst complex and the vesicle by binding phosphatidylinositol 4,5-bisphosphate (PIP_2_) and Rho-family GTPases Cdc42 and Rho3 [40,64,146]. Whereas in fission yeast the whole exocyst complex assembles on vesicles, in the budding yeast, Sec3 localizes to sites of growth independently of vesicles and acts as a pre-localized landmark for incoming vesicles [183].

In summary, Cdc42, once active at the cell tips, binds and activates Pak1 involved in modulating its dynamic local behavior and initiates polarized growth by promoting the delivery of new membrane and cell wall remodeling enzymes via For3 and the exocyst (Figure 2B).

**Control of cell polarity by external cues.** Cell polarization must respond to external cues, although the molecular mechanisms that transduce environmental/extracellular signals to the polarity machinery are far from being completely understood. Remarkably, in fission yeast, the environment modulates the localization of active Cdc42 through the stress-activated protein kinase Sty1, homolog of budding yeast MAPK (mitogen-activated protein kinase) Hog1 and mammalian stress-activated protein kinase p38 [184] (Figure 2A). Sty1 activation induces active Cdc42 dispersal from the cell poles independently of its role as an activator of transcription factor Atf1 and Polo Kinase Plo1 [184]. In contrast, SAPK inactivation prevents scattering of the Cdc42 module, allowing polarized growth even in the complete absence of the actin cytoskeleton [184]. Although the molecular mechanisms by which Sty1 regulates this process remain to be elucidated, the finding that recovery of active Cdc42 at the cell tips after MAP kinase inactivation is very rapid [184] suggests that Sty1 may negatively regulate the coupling of the Cdc42 module to cell-polarity landmarks like Tea1–Tea4 or the formin For3 [184]. It has also been shown that reducing the growth rate of fission yeast cells (either by osmotic stress or physical confinement) induces active Cdc42 domains to rapidly oscillate from cell tips around the cell surface, whereas an increase in growth rate improves polar domain stabilization [185]. A candidate screen for suppressors of this behavior in response to osmotic stress reveals that Sty1, For3 and the myosin type V Myo52 are important players [185]. However, Sty1 acts independently of the Cdc42 effector For3 in this response, since active Cdc42 domains remain fully polarized during osmotic stress in *sty1*Δ*for3*Δ mutant compared to the single deleted mutants [185]. Altogether, this evidence suggests that Sty1 activation induced by different stimuli promotes Cdc42 detachment from the cell tips, which might be mediated by Sty1-dependent phosphorylation of its activators Scd1 or Gef1. Interestingly, this MAP kinase-dependent mechanism of regulation of cell polarity has not been observed in budding yeast, as there is no evidence that Hog1 regulates Cdc42 [186]. Future work will be needed to determine whether a similar regulatory circuit exists in metazoan cells.

**Role of other Rho GTPases in polarization.** The role of GTPases other than Cdc42 during polarized growth is less well understood, although the existence of a crosstalk between Cdc42 and Rho1, Rho2 and Rho5 during the synthesis and remodeling of the cell wall should be expected (discussed in further detail in Section 5). Cells require precise coordination between the cell-wall biosynthetic enzymes and the molecular machinery required for actin organization to ensure viability, with Rho1 GTPase function lying at the crossroad between both processes. In fission yeast, Rho1 is the regulatory subunit of β-GS, and switching off *rho1^+^* expression causes the disappearance of the actin cytoskeleton (Figure 2C) [17]. However, the effector(s) involved in this process is/are not known. Coordination between Cdc42 and Rho1 during polarized growth likely occurs, but so far the molecular details of this interaction have not been deciphered.

Interestingly, a close relationship between the signaling pathway involved in the maintenance of cell wall integrity and the polarity landmarks exists in fission yeast. The Cell Integrity MAPK Pathway (CIP) members Rgf1, Pck1, Pck2 and Pmk1 (see Section 5 for a detailed description of the CIP) cooperate to localize the polarity landmarks Tea1, Tea4 and Pom1 at the cell cortex, as there is a decreased polarity factor enrichment at the cell ends in CIP knockouts [147]. Consistently, several CIP defective mutants show a defect in the establishment of NETO [187]. Conversely, the absence of the above polarity landmarks increases the localization of CIP components at the cell tip, which correlates with a rise in the basal activity of the CIP MAPK Pmk1 (Figure 2C) [147]. These findings might suggest functional crosstalk between the CIP and the machinery involved in establishing polarized growth at the cell ends, which awaits further characterization.

As mentioned earlier, Rho3 GTPase plays important roles in polarized growth and exocytosis [40,42,63], and was initially identified through its interaction with formin For3 (Figure 2B) [40]. Later, Rho3 was shown to genetically interact with *sec8* and *exo70*, two conserved components of the exocyst complex [42]. Indeed, Rho3-deleted cells accumulate putative secretory vesicles of ~100 nm at high temperatures [40,42,43]. Rho3 has also been implicated in Golgi/endosome trafficking regulation through its physical and/or functional interaction with adaptin Apm1, a protein of the clathrin-associated adaptor complex AP-1, and the AP-1 accessory protein Sip1 [43,44]. Sip1 recruits Rho3 to its proper cellular localization in the Golgi/endosomes and facilitates its interaction with the AP-1 complex [44]. Thus, Rho3 might stimulate secretion by locally increasing the exocytic apparatus or through Golgi/endosome regulation, and functions redundantly with the Cdc42 pathway in polarized exocytosis (Figure 2B) [45].

### 4.2. Schizosaccharomyces japonicus

*S. japonicus* is a dimorphic yeast and can transition between the unicellular and the hyphae form in response to certain stimuli [11,188,189]. Polarized growth does not cease during hyphal transition, suggesting that strong crosstalk between growth and division occurs during this process [11]. Hyphal induction requires the activity of the Scd1-Scd2-Cdc42 pathway to form elongated cells, with Scd1 playing a significant role [12]. These cells show a strict dependence on actin-based transport and independence of microtubules to proliferate [11]. As in *S. pombe*, deletion of formin For3 leads to loss of actin cables in *S. japonicus*, and the mutant cells are spherical and cannot polarize growth [11]. Hyphal growth in *S. japonicus* is elicited in response to different environmental stresses [11,189,190]. Therefore, it is plausible that MAPK signaling pathways play an essential role in controlling the dimorphic switch in this fission yeast as in other fungi [191], although the detailed molecular mechanisms are far from being understood. Notably, the Stress Activated MAPK (SAPK) pathway constitutively represses the hyphal growth transition [192]. It is tempting to speculate that Sty1 might negatively regulate hyphal development by modulating the activation status of Cdc42, like in *S. pombe* [184].

## 5. Regulation of Cell Integrity by Rho GTPases

Fungal cellular integrity is preserved by a cell wall that is locally remodeled during polarized growth to allow the uniform internal turgor pressure to expand the cell. Overall, growth depends on the polarized secretion of transmembrane cell wall synthases and the secretion of hydrolases to promote cell wall expansion. Ultimately, both processes are under the control of Cdc42 and are expected to be regulated by numerous signaling pathways. Indeed, growing cells must precisely coordinate and adequately respond to events that might compromise their integrity and cause death by lysis.

**Figure 3:**
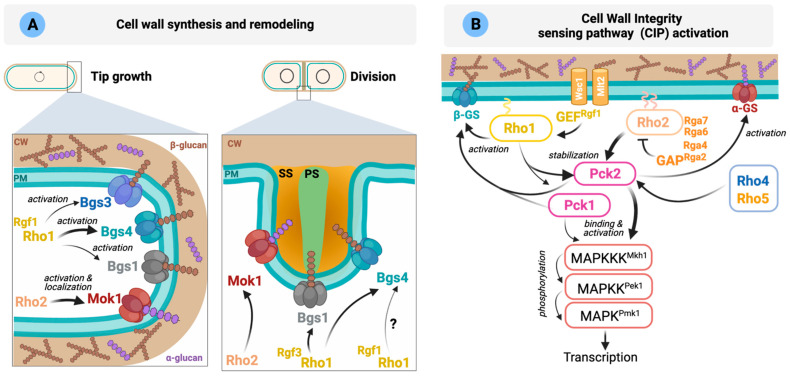
**Rho GTPases involved in the regulation of CW integrity** (adapted from [5,200]). (**A**) Cell wall synthesis at the cell tips during interphase is mediated by α-GS Mok1 (α-glucan) and β-GS Bgs4 (branched β-1,3-glucan). Other β-GS (Bgs1 and 3) also localize to the poles where their role is less well understood. The main function of Bgs1 is the synthesis of the linear β-1,3-glucan of the primary septum, whereas Bgs4 (branched β-1,3-glucan) and Mok1 (α-glucan) synthesize the polymers that form the secondary septum. The Rho GTPases involved in the activation of each GS are indicated in the figure. PM: plasma membrane; CW: cell wall; Glucose units (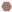,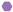); PS: primary septum; SS: secondary septum. (**B**) Schematic representation of *S. pombe* CIP activation. Wsc1 and Mtl2 activate Rho1 through the GEF Rgf1. Rho1 activates the β-GS and stabilizes Pck1 and Pck2. After activation by the phospholipid-dependent kinase Ksg1, both kinases activate the GS. Rho2 regulates Mok1 via Pck2. Rho2-Pck2 are the main activators of the CIP. Please see main text for more details.

**Synthesis and remodeling of the cell wall**. The *S. pombe* cell wall shows a central electron-transparent layer comprised of α-glucans and β-glucans surrounded by two electron-dense layers formed by galactomannan. The septum is also a three-layered structure with a central primary septum made of linear β-1,3-glucan digested upon cell separation, and two outer secondary septa (Figure 3A). A small amount of linear β-1,3-glucan is also present in the cell wall at the tips. However, the majority of the cell wall is composed of branched β-1,3-glucan (reviewed in [5]).

The biosynthesis of β-1,3-glucan is catalyzed by the β-1,3-glucan synthase (GS) complex located in the inner side of the plasma membrane. GTPase Rho1 is the GS regulatory subunit involved in the activation of GS catalytic subunits Bgs1 to Bgs4 in a GTP-dependent manner (Figure 3A). Although not formally demonstrated in fission yeast, it is assumed that Rho1 physically binds to GS catalytic subunits, as it occurs in *S. cerevisiae* [193]. GS activity must be strictly coordinated with the cell cycle, and Rho1 is likely essential to establish this functional link. Indeed, cells lacking Rho1 activity lyse mainly during cytokinesis but also at other cell cycle stages.

PKC orthologs Pck1 and Pck2 are two important Rho1 and Rho2 effectors involved in glucan synthesis [18,20]. Both PKCs share extensive homology at their aminoacidic sequences and have overlapping roles in cell viability [18,37]. They present an extended regulatory domain, including two polybasic coiled-coil HR1 domains that mediate binding and regulation by the GTP-bound Rho GTPases Rho1 and Rho2 [18]. These HR1 domains are closely related to those present in the mammalian Rho family-responsive protein kinase N kinases (PKNs) PKN1-3, a subfamily within the PKC family that binds and becomes regulated by Rho family members [194]. Pck1 and Pck2 are unstable proteins that increase their stability by interaction with the GTPases Rho1 and Rho2 [18,36]. As mentioned earlier, locally active Rho1 regulates the biosynthesis of β-1,3-glucan through direct regulation of the β-GS enzymes, but also indirectly through the activation of Pck1 and Pck2 [18]. Mok1 is the enzyme responsible for synthesizing the cell wall α-1,3-glucan, which is essential for cell integrity [5] and is regulated by Rho2 through Pck2 (Figure 3A) [37]. Cortical actin is required for the localization of these enzymes at the cell poles [5]. Moreover, the correct transport/recycling to the plasma membrane of the GS Bgs1 requires Cdc42 function [195].

How Rho1 activation is precisely controlled in response to internal or external cues to execute its functions remains an unsolved question. Rgf1 GEF specifically activates Rho1 during polarized growth, and its localization to the poles depends on actin and phosphoinositides [31,196]. It activates the β-GS complex containing the catalytic subunit Bgs4 and is involved in NETO [19]. Rgf2 also interacts with Rho1, although Rgf2-deleted cells behave similarly to wild type during polarized growth [27]. Deletion of both Rgf1 and Rgf2 is synthetically lethal, suggesting that both proteins are functionally redundant during vegetative growth [27]. Rgf3 regulates cell wall biosynthesis and cell integrity through Rho1 specifically during septation, and its absence elicits a temperature-sensitive lytic phenotype (Figure 3A, discussed in more detail in Section 6) [25,197]. Rho1 activity is counteracted by Rga1 and Rga5 GAPs throughout the cell cycle. *rga1* null cells show a severe defect in cell growth and a swollen, multiseptated or branched shape [103], a phenotype similar to cells expressing a dominant-active Rho1 mutant [98]. They also show an abnormally thick cell wall and increased the number F-actin patches distributed throughout the cortex [103]. Rga5 cooperates with Rga1 to downregulate Rho1-dependent β-(1,3)-GS activity, and it is also important for the regulation of the other known Rho1 effectors, Pck1 and Pck2. Indeed, Rga5 negatively modulates the Rho1-Pck1, and to a lesser extent Pck2, interaction, causing their destabilization [124]. Since several GEFs and GAPs participate in Rho1 cycling, it is likely that their spatiotemporal regulation might guarantee the precise control of local Rho1 activation during cellular growth. Future studies are needed to fully elucidate the molecular mechanisms underlying the accurate control of Rho1 activity by its GEFs and GAPs.

**Sensing and transducing stimuli through the Cell wall Integrity Pathway (CIP).** Cells can sense changes in the cell wall or membrane caused by different stressors or antifungal drugs and organize an adaptive response critical for survival. In fission yeast, the sensors Wsc1 and Mtl2 play this important role and complement each other to support cell viability [188] and are recruited to different areas: Mtl2 around the cell surface while Wsc1 is enriched at cell tips and also to the septum during cell division [198]. Wsc1 forms stable clusters upon mechanical stress in the cell wall which can serve as local signaling platforms to recruit and activate downstream signaling elements to sites of mechano-perception (Figure 3B) [199].

Both sensors physically couple the cell wall with the plasma membrane to activate Rho1 by two different networks [198]. One involves signaling from Mtl2 through Rho1 to Pck1, while the second one implicates specific signaling from Wsc1 and Rgf2 (and probably also Rgf1) through Rho1 to activate GS and cell wall biosynthesis (Figure 3B). However, Wsc1 and Mlt2 may act independently of the CIP, since CIP signaling is not compromised in *mtl2*Δ and *wsc1*Δ disruptants exposed to cell wall stress, suggesting that the functional relevance of *S. pombe* sensor-like proteins in the CIP pathway differs from that of *S. cerevisiae* [198]. In the latter, the cell surface sensors Wsc1-3, Mid2 and Mtl1 transmit cell wall stress to Rho1 through a set of GEFs and are responsible for activation of the Cell Wall Integrity MAPK pathway [201].

Cell wall homeostasis is regulated by the CIP, which is conserved in all fungi and shows a high degree of similarity to mammalian MAPKs of the ERK1/2 and ERK5 types (Figure 3B) (reviewed in [5,202]). As discussed above, in *S. pombe*, GTP-bound Rho1 and Rho2 interact with and stabilize the two redundant fission yeast PKC orthologs Pck1 and Pck2 [18]. Phosphoinositide-dependent kinase (PDK) ortholog Ksg1 mediates the in vivo activation loop phosphorylation of Pck1 and Pck2 during vegetative growth and stress [22,23]. Rho2 and Pck2 are the primary activators of the MAPK module of the CIP, which is composed of Mkh1 (MAPKKK), Pek1 (MAPKK), and MAPK Pmk1 (Figure 3B). No Rho2 GEFs have been identified so far, but Rho2 activity is downregulated by the GAPs Rga2, Rga4, Rga7 and Rga6, to reduce Pmk1 activation [105]. Rho1 and Pck1 also contribute to Pmk1 activation during vegetative growth and under cell wall perturbations (Figure 3B) [21]. The GEF Rgf1 regulates Pmk1 activation by a mechanism that relies upon both Rho1 and Pck2. Rho1 GEFs Rgf2 and Rgf3 can activate the CIP when overexpressed, but it is not clear if they perform this role at physiological conditions [24]. Whether these GEFs are post-translationally regulated to modulate CIP signaling deserves future work. For instance, in budding yeast, it has been suggested that Mpk1 activates a negative feedback loop that downregulates pathway signaling by depriving Rho1 of its GEF Rom2 [203].

The flow of phosphoinositides and the dynamic lipidation of Rho1 and Rho2 are essential to modulate their membrane localization and the activity of the CIP [33,204,205]. Manipulation of phosphatidylinositol-4-phosphate 5-kinase Its3 function has shown that Pmk1 activity correlates with cellular phosphatidylinositol 4,5-bisphosphate (PI(4,5)P_2_) levels [204]. PI(4,5)P_2_ forms clusters during osmotic stress that co-localize with Rho2; however, Rho2 still forms clusters in the absence of Its3 function, suggesting that Rho2 localization does not require PI(4,5)P_2_. On the other hand, in vivo farnesylation of Rho2 Cys^197^ by farnesyltransferase Cpp1 is critical for membrane binding and downstream signaling to the CIP [34]. During the prenylation process, the free carboxyl group of the isoprenylated cysteine becomes methylated by a specific isoprenylcysteine-*O*-carboxylmethyltransferase (ICMT) [206]. In fission yeast, the absence of ICMT Mam4 prompts a reduction of Rho2 targeting to the plasma membrane, but not in the localization of Rho1 and Cdc42 [205,207]. Rho2 also undergoes in vivo palmitoylation at Cys^196^ by the DHHC palmitoyltransferase Erf2 to mediate its full plasma membrane localization, and this lipid modification is required for morphogenesis control and signaling to the CIP [33].

Rho4 and Rho5 act in addition to Rho2 upstream of the CIP during vegetative growth (Figure 3B) [51]. Both overexpression and deletion of Rho4 or Rho5 affect Pmk1 phosphorylation, and they associate with Pck2, the critical upstream regulator of the CIP MAPK module [51]. However, the fact that a significant fraction of both GTPases localizes to internal membranes in addition to the plasma membrane suggests that Rho4 and Rho5 may control Pmk1 signaling indirectly and through yet unidentified target(s) [51]. Conversely, Rho3 is a negative regulator of the CIP [33]. Accordingly, cells lacking Rho3 exhibit increased Pmk1 activity and strong chloride sensitivity, which is associated with CIP hyperactivation, although this control is independent of the Rho2 function [33].

In summary, Rho2 is the main activator of the CIP, whose activity can be reinforced by additional GTPases such as Rho1, Rho4 and Rho5 that might contribute to modulate pathway activation at specific sites or under specific circumstances.

One important difference between the functional roles of the CIP in budding and fission yeast resides in the control of the actin cytoskeleton. In *S. cerevisiae*, Rho1 is important for proper actin cytoskeleton organization at least through control of the Cell Wall Integrity MAPK Mpk1 [201]. So far, such a functional link has not been established in fission yeast, where the regulatory mechanisms underlying actin cytoskeleton reorganization and integrity in response to different environmental stimuli are mostly unknown. Future studies in *S. pombe* will help to elucidate the details of the possible functional relevance of Rho1 and the CIP in actin organization.

While Pmk1 activity might be positive for maintaining cellular integrity, its constitutive hyperactivation can be deleterious. For instance, inactivation of Pmk1 improves the growth of a *rho1-596* thermosensitive strain which displays severe cell wall defects and high Pmk1 phosphorylation [208]. On the other hand, a recent study has shown that constitutive activation of the CIP in cells grown in heavy water (D_2_O) causes gross morphological changes, thickened cell walls and aberrant septa that affect growth [209]. Remarkably, blocking CIP activity partially alleviates the above phenotypes while activating it increases the D_2_O sensitivity [209]. Therefore, the activity of the CIP must be precisely regulated in time and space to preserve cell wall integrity and cellular viability.

Little is known about the putative functional role of the CIP and its upstream regulators, the Rho GTPases Rho1 and Rho2, and PKC orthologs Pck1 and Pck2 in *S. japonicus*. As in *S. pombe*, both kinases likely regulate the activation status of Pmk1, and also modulate hyphal differentiation dynamics (our unpublished results). Future studies will be required to uncover the role of Rho GTPases in regulating the cell integrity in this dimorphic yeast.

**Interplay between polarized growth and cell wall integrity**. The cell wall gets thinner during polarized cell growth. Thickness at growing tips exhibits a fluctuating behavior with thickening phases followed by thinning stages, indicative of delayed feedback promoting thickness homeostasis. Mechanosensing through the CIP mediates this feedback, which probes tension in the wall to adjust synthase localization and activity to surface growth [133]. Indeed, in the absence of Wsc1, Rgf1 and Pmk1, cell wall thickness homeostasis is severely impaired, which might explain the lytic phenotype observed in some CIP mutants during vegetative growth due to excessive thinning [133]. Since polarized growth might be counteracted by cell wall stiffness, functional crosstalk between the Cdc42 polarity network and the CIP would be expected (Figure 2C). Active Cdc42 promoting polarized growth might modulate the extent of CIP signaling locally. Interestingly, the Cdc42 effector Pak2 shows a two-hybrid interaction with Mkh1, and a dominant-activated *cdc42G12V* allele prompts a growth defect that is rescued by the *pak2*∆ and *mkh1*∆ mutations. This suggested that Cdc42 might signal through the CIP pathway via Pak2 [78]. Unexpectedly, neither *pak2^+^* deletion nor *cdc42G12V* overexpression had a noticeable effect on Pmk1 activation [210]. These findings suggest a scenario in which the Cdc42 polarity pathway and the CIP might coordinately regulate morphogenesis through at least one common target in fission yeast. Another possibility is that the Cdc42 polarity network might regulate Pmk1 activity at a small pool of cell tip localized molecules causing subtle differences difficult to detect. Future studies are necessary to clarify these possibilities. Alternatively, Cdc42 might modulate the cell wall by affecting the activity of Rho GTPases and their direct effectors independently of their role in CIP signaling. In *S. cerevisiae*, a functional relationship between the polarity machinery and Rho1-Pkc1 exists. Laser-induced cell wall and membrane damage results in the Pkc1-dependent disassembly of bud site polarity. This response is expected to facilitate the healing process by providing factors available for cell wall/membrane repair [211]. Rho1 activated Pkc1 promotes the destruction of formin Bni1 and exocyst component Sec3, therefore disrupting polarity complexes, and this degradation is required for the subsequent healing response that includes the recruitment of Pkc1, Rho1, formin Bnr1, Exo70, type V myosin Myo2 and cell wall synthase Chs3 to the site of damage. Whether the GTPases Rho1 and Rho2 modulate the polarity module when the cell wall or the membrane is damaged in fission yeast deserves future investigation.

## 6. Rho Signaling in Cytokinesis

### 6.1. Schizosaccharomyces pombe

**Figure 4:**
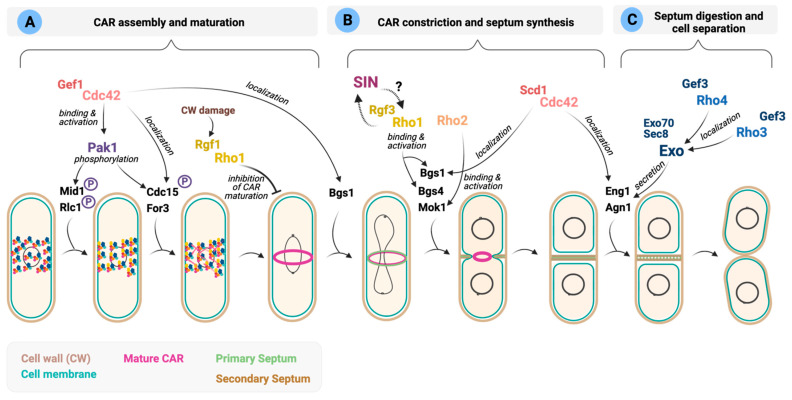
Rho signaling during cytokinesis. Schematic representation of fission yeast cytokinesis. (**A**) CAR precursors are assembled into nodes (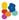) composed of multiple proteins localized into the cell middle by anillin-like protein Mid1. After synthesis of the F-actin network by formins Cdc12 and For3, nodes condense into the CAR through actomyosin interactions. The fully formed ring matures by recruitment of additional cytokinesis proteins (mature car is indicated in magenta). Cdc42 effector Pak1 promotes phosphorylation of CAR components Mid1, Cdc15 and Rlc1. Cdc42 mediates Bgs1 recruitment at the ring during anaphase. Rho1 GTPase participates in a cytokinetic checkpoint that delays CAR maturation after cell wall damage. (**B**) CAR maturation is followed by constriction and cell wall deposition (primary septum, in green, and secondary septum, in brown), which facilitates proper ring closure. Rho1 and Rho2 bind and activate the enzymes involved in septum synthesis. Question mark denotes that the molecular mechanism by which SIN might activate Rho1 remains unsolved. (**C**) Cells separation occurs by digestion of the primary septum and the surrounding cell wall by Agn1 (endo-α-1,3 glucanase) and Eng1 (endo-β-1,3 glucanase). Secretion of Agn1 and Eng1 depends on the activity of Rho4 (activated by Gef3). Both enzymes are targeted to the septum by the exocyst subunits Sec8 and Exo70 regulated by Rho4 and Rho3. Agn1 and Eng1 distribution at the ring is influenced by Cdc42. Please see main text for more details.

In *S. pombe,* polarized growth ceases as cells enter into mitosis and divide. At this stage, the cytoskeletal machinery involved in polar growth during interphase shifts to the cell middle to form the contractile actomyosin ring (CAR) (Figure 4). Most animal and fungal cells, including *S. japonicus*, start to assemble the CAR when chromosomes are fully segregated [84]. In contrast, in *S. pombe*, CAR assembly starts early in anaphase. The interphase nodes containing the anillin-like protein Mid1 and cell cycle kinases evolve into cytokinesis nodes by adding myosin II (Myo2), IQGAP Rng2, F-BAR protein Cdc15, and the formin Cdc12. Cdc12, with help from For3, stimulates the nucleation of actin filaments (Figure 4). Nodes condense to form the CAR by the interactions of myosin II with actin filaments. During the maturation phase, Cdc15 and several other proteins, including Rga7 (Rho2 GAP) and Rgf3 (Rho1 GEF) move to the ring (Figure 4) [212]. Then, the CAR constricts by the action of motor Myp2, another type II myosin, and cofilins, which cut actin fragments stochastically, shortening the ring [212]. The Septation Initiation Network (SIN), a Hippo-related signaling pathway, coordinately triggers constriction of the CAR and the synthesis of new cell walls to form a septum that generates the physical barriers that separate the two daughter cells [213]. Finally, cell separation liberates two daughter cells upon dissolution of the primary septum by the action of glucanases Eng1 and Agn1 delivered to the division site (Figure 4) [214].

In *S. pombe*, the Rho GTPases, Cdc42, Rho1, Rho3 and Rho4 have been reported to control different steps in cytokinesis (Figure 4) (recently reviewed in [6,88]). In contrast, in *S. japonicus,* the putative roles of the Rho GTPase signaling network during cytokinesis have not been addressed.

**Cdc42 regulates early and late cytokinetic events.** The role of Cdc42 in polarized growth has been well documented; however, less is known about its function during cytokinesis. Several reports have suggested that Cdc42 regulates the recruitment of specific cargoes required for distinct steps during division [86,195,215].

In fission yeast, the successive recruitment to the division site of GEFs Scd1 and Gef1 activates Cdc42 locally [86]. Gef1 localizes first to the membrane proximal to the actomyosin ring where it activates Cdc42 to promote Bgs1 recruitment, and the timely onset of ring constriction and septum ingression (Figure 4A). However, the precise molecular mechanism involved in Bgs1 delivery remains unclear [86]. This is followed by Scd1 localization to the division site in a Gef1-dependent manner to activate Cdc42 and promote Bgs1 localization along the ingressing furrow and efficient septum formation (Figure 4B) [86]. Scd1 itself restricts Gef1 localization from the division site [74]. Cdc15 also contributes to Gef1 localization to the cell tips and the division site, although the molecular details are unknown [75]. Reciprocally, Gef1-activated Cdc42 promotes endocytic events that prompt uniform Cdc15 assembly at the ring and concentric furrow formation [90].

The Cdc42 effector Pak1 has a prominent role in fission yeast cytokinesis. Pak1 colocalizes with the essential class-II myosin Myo2 in the contractile ring, where it phosphorylates the myosin regulatory light chain Rlc1 [81]. Such phosphorylation may serve to slow node dynamics by reducing force production by Myo2, which permits sufficient time to allow chromosome segregation to occur before cytokinesis [87]. Pak1 also phosphorylates and regulates the localization to the CAR of other cytokinetic proteins like the contractile ring anillin-related protein Mid1, F-BAR protein Cdc15, and Cyk3 (Figure 4A) [89,92]. In budding yeast, PAK kinases have not been involved in the phosphorylation of Cdc15 ortholog Hof1, which depends primarily on cell cycle kinases [216]. Instead, Cla4, a PAK family member, regulates the selection of the cell division site by engaging the positive feedback loop by phosphorylation of Cdc42 activators [217] and by direct phosphorylation of septin filament subunits Cdc2, Cdc10, Cdc11 and Cdc12 [218].

The formin For3 is another Cdc42 downstream effector that localizes to the division site shortly after spindle pole body (SPB) separation during CAR assembly (Figure 4). [83]. *for3*Δ cells show a delay in the later stages of CAR constriction and disassembly compared to wild-type cells [83]. Accordingly, For3 nucleated actin cables are critical for efficiently delivering secretory vesicles carrying new membrane by type V myosin Myo52 to the division site [82,85]. For3 function becomes essential during actin assembly for node movement and cell survival in mutants with reduced formin Cdc12 activity, suggesting that both formins cooperate to nucleate actin filaments for CAR assembly and disassembly during cytokinesis [83]. Remarkably, the SAPK pathway and its effector, MAPK Sty1, elicit CAR disassembly in *S. pombe* when its integrity becomes compromised during actin damage and stress by downregulating For3 levels, thereby blocking cell division until the damage is repaired. The molecular mechanism that controls For3 under these circumstances seems Cdc42 independent, but its participation cannot be completely ruled out [91].

The role of Cdc42 during the final stages of cytokinesis is not clearly understood. Cdc42 activity is required for proper localization at the ring of the septum digesting glucanases Agn1 and Eng1 (Figure 4C) [215]. However, Cdc42 needs to be inactivated to complete cytokinesis since cells overexpressing a constitutively active Cdc42 allele form a septum but fail to undergo cell separation [4,6]. It seems plausible that the activity of Cdc42 must be strictly temporally controlled during septum dissolution because either lack or excess of GTPase activity induces separation defects. Cdc42 GAPs Rga3, Rga4 and Rga6 might contribute to achieving such control since they all localize to the division site [73,121,122,125].

**On the role of other GTPases in the control of late cytokinetic events.** The role during cytokinesis of other Rho GTPases besides Cdc42 has been mainly associated with the last stages of septum synthesis and dissolution.

The knowledge on the putative function/s of Rho1 during the early steps of cytokinesis is scarce, and its main role during this process seems intimately connected to its function as an activator of the GS complex (Figure 4B). Rho1 regulated β-GSs Bgs4 and Bgs1 contribute to keeping the CAR in position until constriction starts [219,220]. In contrast, RhoA and Rho1 in animal cells and budding yeast are active in early cytokinesis for division-site selection and contractile ring assembly, respectively [221,222].

The cytokinetic role of several Rho1 regulators that interact physically with CAR components has been also depicted. The F-BAR scaffold Cdc15 contains an SH3 domain that binds proteins required for septation, such as paxillin Pxl1 or Rgf3. Pxl1 negatively modulates Rho1 activity and interacts with Bgs1 for the stable anchorage of the CAR to the plasma membrane and furrow formation [29,223]. As discussed in Section 5, Rgf3 is an essential GEF that activates Rho1 at the CAR (Figure 4B) [26]. Lack of function of Rgf3 delays ring maturation and constriction [27,119], and cells lyse during cell division when the primary septum is degraded [26]. Rgf3 localization also depends on the arrestin Art1. In *art1*∆ cells, low levels of Rgf3 at the division site lead to inefficient Rho1 activation, causing septum defects and cell lysis due to poor activation of β-GSs [119]. The fact that Art1 does not affect Bgs1 localization suggests that Rho1 plays no prominent role in Bgs1 localization, which has been shown to rely on the exocyst complex, Cdc42 and F-BAR protein Cdc15 [64,220]. Recently, it has been described that Rgf3 is more concentrated at the center of the division site in the absence of the UNC-13/Munc13 protein Ync13, which correlates with an elevated amount of active Rho1 at the division site [224]. This abnormal protein distribution is caused by defective site selection during endocytosis, for which Ync13 is relevant. As expected, Bgs1, Bgs4 and Mok1 localize abnormally at the CAR in Ync13 null cells [224]. Given the essential role of Rgf3, the identification of its upstream regulators will surely help to understand how Rho1 regulates cell wall integrity during fission yeast cytokinesis. On the other hand, Rgf1, the main GEF that activates Rho1 at the growing ends during polarized growth, is also functionally relevant during cytokinesis [31]. Rgf1 and Rho1 participate in a checkpoint that imposes a delay in the maturation phase of CAR assembly upon stress affecting cell wall integrity [31]. This inhibition is exerted through Rgf1-Rho1 activation of the CIP components Pck2 and Pmk1 to ensure that cytokinesis is completed after cells have adapted to the new conditions (Figure 4A) [31].

Recent work has shown that the amount of active Rho1 at the division site is also influenced by astral microtubules. Rho1-GTP signal peaks during furrow ingression and cell separation, and this accumulation is enhanced in lack of function mutants in the gamma-tubulin complex linkers Mto1 and Mto2 [225]. Despite these initial pieces of evidence, it is still unclear which Rho1 regulators contribute to this hyperactivation, how microtubules are connected to these proteins, and, most importantly, how increased Rho1 accumulation influences furrow ingression.

Rho2 GTPase localizes to the septum during cytokinesis, and induces a strong cell separation defect upon overexpression [32]. However, the specific role of this GTPase during cytokinesis is unknown. Rho2 activates the α-GS Mok1 through the protein kinase C (PKC) homologue Pck2 [18,35]. This might contribute to proper septum formation and dissolution, given that Mok1 is essential for the adequate synthesis of both the primary and secondary septa and to support the physical forces of the cell turgor pressure during cell separation (Figure 4B) [39]. In addition, Rho2 acts upstream the CIP MAP kinase pathway [34], which becomes activated during the cell cycle peaking at cell separation during cytokinesis [226]. Indeed, loss of Pmk1 activity or its hyperactivation leads to cell separation defects [202]. This cytokinetic defect might be related to Pmk1 dependent phosphorylation of the RNA-binding protein Nrd1, which is involved in stabilizing *myo2^+^* mRNA [227]. Alternatively, Pmk1 might be necessary for the local activation of the phosphatase calcineurin at the CAR, where it is targeted by Pxl1 and participates in the dephosphorylation of the F-BAR protein Cdc15 [228]. However, Pmk1 phosphorylation during cytokinesis is not dependent on Rho2 [38,202], and therefore it is not clear whether Rho2 has a relevant function during this process.

Rho5 GTPase also localizes to the division site, and its activity is redundant with that of Rho1 [52]. Cells overexpressing *rho5^+^* exhibit morphological defects and have an elevated septation index; therefore, suggesting a role during septum formation or ring stability. GEF Gef2 confers stability to the actomyosin ring, and it is an in vitro binding partner of Rho1, Rho4 and Rho5 [101,102]. Thus, Rho1/5-dependent GS activity might be required to stabilize the actomyosin ring [6].

The SIN pathway is essential for fission yeast CAR contraction and simultaneous septum synthesis (reviewed in [229]). In the absence of SIN signaling, cells do not initiate septum synthesis, resulting in the formation of elongated multinucleated cells [229]. It has been proposed that SIN activates Rho1, which in turn activates the Bgs enzymes [28], and that there is a feedback loop where active Rho1 stimulates the SIN while septation is progressing (Figure 4B) [30]. How these regulatory interactions occur at the molecular level is currently unknown. One possibility is that the SIN targets Rho1 by activating Rgf3 [28].

The final and most critical step of cytokinesis is cell separation, which initiates by the activation of the Morphogenesis Orb6 (MOR) pathway upon completing actomyosin ring constriction and septum formation. In fission yeast, the separation machinery includes septin protein complexes (Mid2 and septins Spn1-4), the exocyst, Rho GTPases and the glucanases Eng1 and Agn1 (reviewed in [230]). First, Agn1 digests the cell wall material that surrounds the septum followed by the action of Eng1, which is necessary for the dissolution of the primary septum. Both proteins localize to a ring-like structure surrounding the septum, which depends on the exocyst components Sec8 and Exo70, the septins and Mid2. The localization of the septin ring and the exocyst complex at the division site requires Rho4 GTPase [50] (Figure 4C). Likewise, both complexes are needed for GEF Gef3 localization which in turn activates Rho4 [45,107]. Rho4 levels are reduced, and the localization of Eng1 and Agn1 glucanases is abnormal in cells lacking Gef3. Gef3 also interacts with GTPase Rho3 that participates in cell separation by modulating exocyst function (Figure 4C) [45].

### 6.2. Schizosaccharomyces japonicus

*S. japonicus* breaks down the nuclear envelope during mitosis and assembles the actomyosin cytokinetic ring only after the exit from mitosis through a Cdc15-dependent ring anchorage system relying on cell tip-localized cortical cues [84,231]. Forcing *S. japonicus* cells to divide at smaller volume by reducing Cdk1 activation status at G2/M either genetically or nutritionally, leads to re-scaling of cellular geometry transitioning through an asymmetrically dividing stage [232]. During this process, the spatial regulation of Cdc42 activity by Rga4 GAP becomes critical, and contributes to proper patterning of the cortical domains required for division site placement [232].

In *S. japonicus,* the anaphase nucleus controls the spatial distribution of actin nucleation. However, it is currently unknown whether the signal is released from the nuclear compartment or derives from a nuclear structure such as the SPB or the mitotic spindle. Recent findings suggests that actin nucleation during CAR assembly depends mostly on formin Cdc12, although For3 also cooperates when Cdc12 activity is compromised [83]. Although there is no evidence that Cdc42 controls Cdc12-dependent actin nucleation in *S. pombe* [233], it does play an essential role in the activation of For3 [60]. It will be interesting to investigate the dynamics of *S. japonicus* Cdc42 activation at the equatorial cortex and possible links to Cdc12 and For3 activation.

## 7. Functions of Rho GTPases during Sexual Differentiation

**Figure 5:**
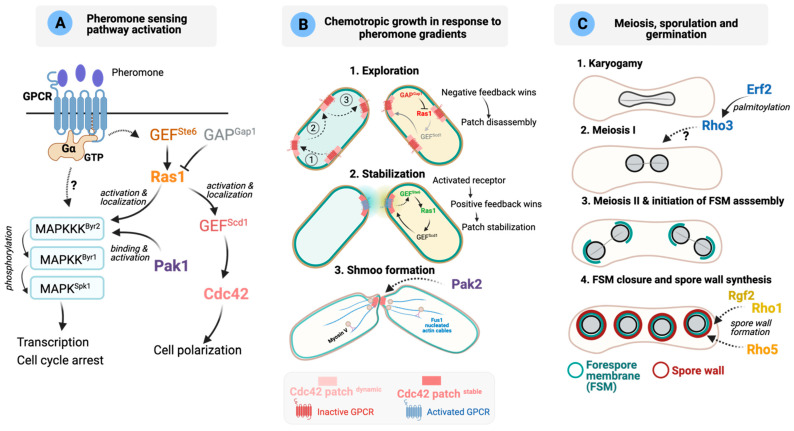
Functions of Rho GTPases during sexual differentiation (adapted from [7]). (**A**) GPCR (G-protein-coupled receptors) activation promotes MAPK signaling by the Gα Gpa1 and the Ras GTPase. Ras1 also promotes Cdc42 activation for polarization during mating. (**B**) Cells form dynamic polarization zones that contain pheromone release and perception machinery that probe the environment for partner choice (exploration). The dynamic behavior requires patch destabilization promoted by negative control on Ras1. These zones are stabilized by opposite-type pheromones (represented in blue and yellow semicircles). Higher pheromone sensing promotes Gα activation, which leads to enhanced Ste6 and Ras1 activation and patch stabilization. Upon cell–cell contact, cell wall remodeling allows plasma membrane contact, fusion pore formation and pore expansion for zygote formation. Fus1 is a formin essential for fusion and for actin localization at the shmoo tip. The type V myosins serve to concentrate the cell wall glucanases to drive local cell wall dissolution for cell fusion. (**C**) Diagrammatic representation of sporulation stages in *S. pombe*. After cell pairing and fusion, the two nuclei fuse as well (karyogamy) and the resulting diploid nucleus undergoes meiosis. The zygote develops into an ascus, containing four dormant ascospores. The initiation of forespore membrane (FSM) assembly occurs during meiosis II. Shortly after, FSM extension encapsulates nucleus and cytoplasm and closure occurs. Finally, spore wall is synthesized. The role of several Rho GTPases during the different stages is shown in the figure. Dotted arrows mean that the molecular links have not been established. See main text for more details.

The strong relevance of the cytoskeleton in polarized growth during mating suggests a requirement for Rho GTPases function [234]. In particular, Cdc42 is essential for polarization in response to external pheromone gradients during *S. pombe* sexual reproduction (recently reviewed in [7]), a process where cells of the opposite mating type plus (P or h^+^) and minus (M or h^−^) pair during mating [235,236]. The binding of pheromone receptors Map3 (h^+^) [237] and Mam2 (h^−^) [238] with their correspondent pheromone ligands (M and P factors) stimulates the activation of a receptor-associated heterotrimeric G-protein. Subsequently, activated Gpa1 α subunit signals to the conserved mating-pheromone responsive MAPK module composed by MAPKKK Byr2, MAPKK Byr1, and MAPK Spk1 (Figure 5A) [239,240], functionally homologous to the mammalian Ras-Raf-MEK-ERK mitogenic pathway [241]. So far, the molecular links between the Gα and the MAPKKK Byr2 remain unknown. The connections between the G proteins and the MAPK cascade are better understood in *S. cerevisiae*. Pheromone receptor activation releases Gβγ that activate the MAPK cascade to induce cell differentiation. Gβγ also associates with the GEF Cdc24, which activates Cdc42, and with Rho1, mediating its proper localization to the tip of the mating projection [242]. In fission yeast, the small GTPase Ras1 is another regulator of the MAPK cascade. Ras1 activation by Ste6 recruits Byr2 to the plasma membrane and relieves its autoinhibition, thereby causing Byr2 to drive sequential phosphorylation of Byr1 and Spk1 (Figure 5A) [243,244]. MAPK cascade components modulate the expression of mating-specific genes that activate the transcription factor Ste11 to induce the differentiation process (Figure 5A) [245,246].

**Cdc42 signaling during sexual differentiation.** Cdc42 signaling plays a critical role during mating by contributing to MAPK activation through one of its main effectors, the essential kinase Pak1, which mediates the transition of MAPKKK Byr2 to an activated state (Figure 5A) [247]. Surprisingly, the Cdc42 GEF Scd1 and the scaffold protein Scd2, which promote Cdc42 activation and are critical during sexual differentiation, are not required for MAPK activation, suggesting that the mating defect of cells lacking Scd1 or Scd2 is primarily due to their inability to perform polarized cell growth [57]. It might be possible that residual GTPase activity prompted by the action of Gef1, the other Cdc42 GEF, may be sufficient for Pak1 activation, but not for polarized cell growth, thus resulting in sterility [246]. In contrast, in budding yeast, the scaffold protein Bem1 (ortholog to Scd2) binds the PAK kinase Ste20 and the MAPK scaffold Ste5 [248], to recruit components of the MAPK to the shmoo site.

**Formation of the mating projection and cell pairing.** In addition to MAPK signaling during sexual differentiation, Cdc42 function is also critical in the process of polarized morphogenesis in the direction of the partner cell (shmooing). Cdc42-GTP forms dynamic zones or patches before cell pairing that explore the cell cortex upon pheromone exposure, independently of the direction of the pheromone gradient [93,94]. Patches containing activated Cdc42 reorient over time, stabilize toward the chosen pheromone-secreting partner, and start growing towards it via Cdc42-dependent recruitment of the cell growth machinery (Figure 5B) [93,94,96]. Pheromones are locally released in these patches, as they become enriched in Gpa1 α subunit, suggesting that they represent receptor activation sites [7,94]. As a whole, this strategy helps the whole cell population to explore space for possible mating configurations, thus optimizing the population mating efficiency [94]. In budding yeast, in contrast, cells form a Cdc42-GTP patch that wanders around the cell cortex, executing a biased random walk rather than appearance and disappearance [7].

GTP-Cdc42 patch lifetime depends upon positive and negative feedbacks, whereby negative signals promote its disassembly and a local increase in the positive feedback, which could occur by local pheromone sensing, stabilizes the patch against competition from other patches (Figure 5B) [96]. During pheromone-dependent polarization, Rga3 is recruited to Cdc42 patches, where it promotes their exploratory dynamics, limits projection growth in response to pheromone, and confers a competitive advantage during sexual reproduction [73]. Thus, during mating Rga3 may form negative feedback, encouraging active Cdc42 zone turnover. However, cells lacking all Cdc42 GAPs retain almost complete ability to polarize and mate during sexual differentiation, despite lacking growth polarity during mitotic growth [73]. This suggests that GTP-Cdc42 dependent polarization may depend on other modes of negative regulation during mating. In fact, it has been shown that patch assembly/disassembly dynamics rely on Ras1, which localizes at the same exploratory sites as active Cdc42 [96]. Patch destabilization is promoted by the negative control caused by the recruitment of GAP Gap1 by activated Ras1 (Figure 5B) [96]. Nevertheless, cells lacking the Ras1 GAP still can form unstable patches at low pheromone concentrations, suggesting that it is not the sole patch destabilization mechanism [95]. This negative feedback control of Ras activity is also responsible for restraining the MAPK signal, and couples fusion with cell–cell engagement [95]. On the other hand, patch stabilization upon pheromone sensing depends on Gα Gpa1, which is the sole signal transducer in fission yeast, and promotes the localization and/or activation of the Ras GEF Ste6 to overcome the negative control by the Ras GAP Gap1 [7]. Pheromone-induced expression of Ste6 is itself regulated by Ras1 activity, contributing to the positive feedback loop (Figure 5B) [243,249].

Recent data have revealed an essential role for the Cdc42 effector kinase Pak2 during cell pairing. In the absence of Pak2, cells show many unfused partners and a prolonged lifetime of the actin fusion focus (Figure 5B) [250]. Moreover, *pak2*Δ matings produced about 10% aberrant asci formed upon meiosis and sporulation in homothallic haploid cells [250]. Rho3 is also important during the meiotic process, during which it becomes increasingly palmitoylated by the Erf2 DHHC-palmitoyltransferase (Figure 5C). Rho3 palmitoylation is vital for meiotic entry, since Erf2 overproduction in proliferating cells induces a meiotic phenotype that requires the activity of this GTPase [251].

**Spore formation and germination**. Spores are created after each of the four haploid nuclei produced by meiosis are packaged into daughter cells by envelopment within newly synthesized membranes called forespore membranes (FSM). Forespore membrane formation initiates on meiotic spindle pole bodies early in meiosis II. Then, each forespore membrane expands to engulf the associated nucleus, after which these cells mature into spores by deposition of spore wall material. All of these events occur within the cytoplasm of the original mother cell, referred to as the ascus (Figure 5C) [252].

Rho1 and Rho5 GTPases are required for proper spore wall formation. A role for Rho1 GEF Rgf2 in spore wall maturation has been proposed, since *rgf2*∆ zygotes produced immature ascospores unable to germinate, a phenotype that might be related to defective Bgs2 activation (Figure 5C) [253]. As noted above, in budding yeast, Rho1 is recruited by pheromone-activated Gβγ subunits to the site of polarized growth [242]. Such a functional link has not been established in fission yeast, where Rgf2 appears later during the differentiation process when the spore outline is visible under phase-contrast microscopy. The GTPase Rho5, a functional paralogue of Rho1, also participates in the spore wall formation. Ascospores lacking Rho5 are less resistant to heat or lytic enzymes than wild-type spores (Figure 5C) [53].

Once conditions are favorable, spores germinate to exit dormancy, resume growth, and develop a single polarized tube that hatches out of the outer spore wall [254]. Active Cdc42 plays an essential role in spore germination by spontaneously polarizing and by forming dynamic zones that stabilize upon spore outgrowth [255].

## 8. Future Avenues

As highlighted in this review, a single Rho GTPase can regulate different cellular processes, depending on the stimulus and the cell type. Therefore, tight and precise spatiotemporal regulation of Rho GTPase function is critical to determine the specific outcome of its activity. Growing evidence demonstrates that phosphorylation may serve as one of the predominant signals controlling the activity, interactions, and localization of Rho GTPases and its regulators by multiple signaling cascades. For instance, mammalian RhoA is phosphorylated close to its CAAX box on Ser^188^ by the cAMP- or cGMP-dependent protein kinase (PKA or PKG, respectively), which negatively regulates RhoA activity by enhancing its interaction with Rho GDI and its extraction (translocation) from membranes, without direct perturbation of GEF, GAP or geranylgeranyl transferase activity [256]. A similar phenomenon has been observed with Cdc42 in vitro [257]. Fission yeast Cdc42 harbors a conserved Ser^187^ which lies at the same position as Ser^188^ in RhoA, whereas Rho1 Ser^191^ is located upstream the C-terminal polybasic sequence. Interestingly, phosphoproteomics studies have shown that Rho1 Ser^191^ might be phosphorylated in vivo [258]. However, to date, there is no evidence that fission yeast Rho GTPases are regulated by direct phosphorylation, either the identity of the signaling pathways involved. Data on the post-translational regulation of GEFs and GAPs functions are also scarce. Recent phosphoproteomic studies have just started to shed light on some of the regulatory pathways that participate in such control, but additional research is required for a more complete knowledge on the post-translational modifications of Rho GTPases and their regulators, and the comprehension of their functional relevance.

The understanding of how eukaryotic cells organize an adaptive response to different environmental/extracellular signals to guarantee survival is of the utmost importance. At the core of these responses are Rho GTPases, which regulate major physiological processes such as polarized growth, morphogenesis, cellular integrity, cytokinesis, and cellular differentiation. Further comparative studies employing fission yeast models will surely help to illuminate novel aspects of the immensely complex regulatory mechanisms encompassing Rho GTPase signaling.

## Figures and Tables

**Table 1:** Rho GTPases in fission yeast.

GTPase	GEF	GAP	GDI	Localization	Regulated/Involved Processes
Rho1 ^(●)^	Gef2 ^(+)^ Rgf1 Rgf2 Rgf3 ^(●)^	Rga1 Rga5 Rga8	Rdi1	Active growing sites (cell poles, division area) [17]	- Morphogenesis and cell polarity [3,17,18,19] - Cell wall biosynthesis by Bgs1-4 β(1,3)-GSs regulation (regulatory subunit) after the membrane sensors Wsc1 and Mlt2 and by Pck1 and Pck2 stabilization (both activated by Ksg1, in turn, activate β-GSs) [3,17,18,19,20,21,22,23] - CIP regulation in response to cell wall damage, independently of Wsc1 and Mlt2 [21,24] - Actomyosin ring stabilization and primary septum formation (cytokinesis) [17,25,26,27,28,29,30] - Cytokinesis checkpoint in response to cell wall damage via Pck2-Pmk1 [31]
Rho2	Rgf1?	Rga2 Rga4 Rga6 Rga7		Active growing sites (cell poles, division area) [32] Modified by palmitoylation [33] and target of farnesyltransferase Cpp1 [34]	- Morphogenesis and cell polarity [18,32,35,36] - Cell wall biosynthesis by activation of Mok1/Ags1 α(1,3)-GS via Pck2 [35,37] - CIP regulation together with Pck2 [34,38] - Mok1 contributes to the primary and secondary septum formation and prevent lysis during cell separation (cytokinesis) [39]
Rho3 ^(●)^	Gef3 ^(+)^			Division area [40]	- General role in secretion [41] by exocytosis [40,42] and/or Golgi/endosome trafficking [43,44,45]: ■ With Rho4 enable cell separation through the delivery and secretion of glucanases Agn1 and Eng1 (cytokinesis) [42] ■ Possible redundant function with Cdc42 in polarity exocytosis and cytokinesis [45] - Sexual differentiation [46]
Rho4	Scd1 Gef2 ^(+)^ Gef3	Rga9	Rdi1	Cell periphery (interphase) and division area (M-phase) [47], septum [48]	- Morphology, septation and cell wall integrity affecting secretion of glucanases [47,48,49,50]: ■ Interacts with the exocyst and is necessary for the correct localization of septins [50] ■ With Rho3 regulates the localization and secretion of glucanases Agn1 and Eng1 during cell separation (cytokinesis) [47,49,50] - Possible role in CIP signaling pathway [51]
Rho5	Gef2 ^(+)^			Cell poles (interphase) and division area (M-phase) [52]	- Functional paralogue of Rho1 [52,53] - Possible role in CIP signaling pathway [51] - Cytokinesis (probably contributes to septum formation or ring stability, similarly to Rho1) - Meiosis and sporulation (expressed during stationary phase) [53]
Cdc42 ^(●)^	Scd1 Gef1	Rga3 Rga4 Rga6 Rga9 ^(+)^	Rdi1	Endomembranes and active growing sites (cell poles, division area) [4,54,55,56]	- Morphogenesis, membrane trafficking, endosome recycling, vacuole formation and cell polarity [4,40,56,57,58,59,60,61,62,63,64,65,66,67,68,69,70,71,72,73,74,75,76,77] - Actomyosin ring and septum formation (cytokinesis) [6,55,74,75,78,79,80,81,82,83,84,85,86,87,88,89,90,91,92] - Pheromone response (mating) and sexual differentiation [54,57,73,93,94,95,96]

^(●)^ Essential gene; *rho3* only at high temperatures [97]. ^(+)^ in vitro binding partner. GS: glucan synthase; CIP: cell integrity pathway.

## References

[1] Hodge R.G., Ridley A.J. (2016). Regulating Rho GTPases and their regulators. Nat. Rev. Mol. Cell. Biol..

[2] Phuyal S., Farhan H. (2019). Multifaceted Rho GTPase signaling at the endomembranes. Front. Cell. Cev. Biol..

[3] Arellano M., Duran A., Perez P. (1996). Rho1 GTPase activates the (1-3)β-D-glucan synthase and is involved in *Schizosaccharomyces pombe* morphogenesis. EMBO J..

[4] Miller P.J., Johnson D.I. (1994). Cdc42p GTPase is involved in controlling polarized cell growth in *Schizosaccharomyces pombe*. Mol. Cell. Biol..

[5] Pérez P., Cortés J.C.G., Cansado J., Ribas J.C. (2018). Fission yeast cell wall biosynthesis and cell integrity signalling. Cell Surf..

[6] Hercyk B., Das M. (2019). Rho family GTPases in fission yeast cytokinesis. Commun. Integr. Biol..

[7] Martin S.G. (2019). Molecular mechanisms of chemotropism and cell fusion in unicellular fungi. J. Cell. Sci..

[8] Klar A.J.S. (2013). *Schizosaccharomyces japonicus* yeast poised to become a favorite experimental organism for eukaryotic research. G3 (Bethesda).

[9] Niki H. (2014). *Schizosaccharomyces japonicus*: The fission yeast is a fusion of yeast and hyphae. Yeast.

[10] Wickerham L.J., Duprat E. (1945). A remarkable fission yeast, *Schizosaccharomyces versatilis* nov. sp. J. Bacteriol..

[11] Kinnaer C., Dudin O., Martin S.G. (2019). Yeast-to-hypha transition of *Schizosaccharomyces japonicus* in response to environmental stimuli. Mol. Biol. Cell.

[12] Nozaki S., Furuya K., Niki H. (2018). The Ras1-Cdc42 pathway is involved in hyphal development of *Schizosaccharomyces japonicus*. FEMS Yeast Res..

[13] Cherfils J., Zeghouf M. (2013). Regulation of small GTPases by GEFs, GAPs, and GDIs. Physiol. Rev..

[14] Bos J.L., Rehmann H., Wittinghofer A. (2007). GEFs and GAPs: Critical elements in the control of small G proteins. Cell.

[15] Liu M., Bi F., Zhou X., Zheng Y. (2012). Rho GTPase regulation by miRNAs and covalent modifications. Trends Cell. Biol..

[16] Croft D.R., Olson M.F. (2011). Transcriptional regulation of Rho GTPase signaling. Transcription.

[17] Arellano M., Duran A., Perez P. (1997). Localisation of the *Schizosaccharomyces pombe* Rho1p GTPase and its involvement in the organisation of the actin cytoskeleton. J. Cell Sci..

[18] Arellano M., Valdivieso M.H., Calonge T.M., Coll P.M., Duran A., Perez P. (1999). *Schizosaccharomyces pombe* protein kinase C homologues, Pck1p and Pck2p, are targets of Rho1p and Rho2p and differentially regulate cell integrity. J. Cell Sci..

[19] Garcia P., Tajadura V., Garcia I., Sanchez Y. (2006). Rgf1p is a specific Rho1-GEF that coordinates cell polarization with cell wall biogenesis in fission yeast. Mol. Biol. Cell.

[20] Sayers L.G., Katayama S., Nakano K., Mellor H., Mabuchi I., Toda T., Parker P.J. (2000). Rho-dependence of *Schizosaccharomyces pombe* Pck2. Genes Cells.

[21] Sanchez-Mir L., Soto T., Franco A., Madrid M., Viana R.A., Vicente J., Gacto M., Perez P., Cansado J. (2014). Rho1 GTPase and PKC ortholog Pck1 are upstream activators of the cell integrity MAPK pathway in fission yeast. PLoS ONE.

[22] Madrid M., Jiménez R., Sánchez-Mir L., Soto T., Franco A., Vicente-Soler J., Gacto M., Pérez P., Cansado J. (2015). Multiple layers of regulation influence cell integrity control by the PKC ortholog Pck2 in fission yeast. J. Cell Sci..

[23] Madrid M., Vázquez-Marín B., Soto T., Franco A., Gómez-Gil E., Vicente-Soler J., Gacto M., Pérez P., Cansado J. (2017). Differential functional regulation of protein kinase C (PKC) orthologs in fission yeast. J. Biol. Chem..

[24] Garcia P., Tajadura V., Sanchez Y. (2009). The Rho1p exchange factor Rgf1p signals upstream from the Pmk1 mitogen-activated protein kinase pathway in fission yeast. Mol. Biol. Cell.

[25] Tajadura V., Garcia B., Garcia I., Garcia P., Sanchez Y. (2004). *Schizosaccharomyces pombe* Rgf3p is a specific Rho1 GEF that regulates cell wall β-glucan biosynthesis through the GTPase Rho1p. J. Cell Sci..

[26] Morrell-Falvey J.L., Ren L., Feoktistova A., Haese G.D., Gould K.L. (2005). Cell wall remodeling at the fission yeast cell division site requires the Rho-GEF Rgf3p. J. Cell Sci..

[27] Mutoh T., Nakano K., Mabuchi I. (2005). Rho1-GEFs Rgf1 and Rgf2 are involved in formation of cell wall and septum, while Rgf3 is involved in cytokinesis in fission yeast. Genes Cells.

[28] Jin Q.-W., Zhou M., Bimbo A., Balasubramanian M.K., McCollum D. (2006). A role for the septation initiation network in septum assembly revealed by genetic analysis of *sid2-250* suppressors. Genetics.

[29] Pinar M., Coll P.M., Rincon S.A., Perez P. (2008). *Schizosaccharomyces pombe* Pxl1 is a paxillin homologue that modulates Rho1 activity and participates in cytokinesis. Mol. Biol. Cell.

[30] Alcaide-Gavilan M., Lahoz A., Daga R.R., Jimenez J. (2014). Feedback regulation of SIN by Etd1 and Rho1 in fission yeast. Genetics.

[31] Edreira T., Celador R., Manjón E., Sánchez Y. (2020). A novel checkpoint pathway controls actomyosin ring constriction trigger in fission yeast. eLife.

[32] Hirata D., Nakano K., Fukui M., Takenaka H., Miyakawa T., Mabuchi I. (1998). Genes that cause aberrant cell morphology by overexpression in fission yeast: A role of a small GTP-binding protein Rho2 in cell morphogenesis. J. Cell Sci..

[33] Sanchez-Mir L., Franco A., Martin-Garcia R., Madrid M., Vicente-Soler J., Soto T., Gacto M., Perez P., Cansado J. (2014). Rho2 palmitoylation is required for plasma membrane localization and proper signaling to the fission yeast cell integrity mitogen- activated protein kinase pathway. Mol. Cell. Biol..

[34] Ma Y., Kuno T., Kita A., Asayama Y., Sugiura R. (2006). Rho2 is a target of the farnesyltransferase Cpp1 and acts upstream of Pmk1 mitogen-activated protein kinase signaling in fission yeast. Mol. Biol. Cell.

[35] Katayama S., Hirata D., Arellano M., Pérez P., Toda T. (1999). Fission yeast α-glucan synthase Mok1 requires the actin cytoskeleton to localize the sites of growth and plays an essential role in cell morphogenesis downstream of protein kinase C function. J. Cell Biol..

[36] Villar-Tajadura M.A., Coll P.M., Madrid M., Cansado J., Santos B., Perez P. (2008). Rga2 is a Rho2 GAP that regulates morphogenesis and cell integrity in *S. pombe*. Mol. Microbiol..

[37] Calonge T.M., Nakano K., Arellano M., Arai R., Katayama S., Toda T., Mabuchi I., Perez P. (2000). *Schizosaccharomyces pombe* Rho2p GTPase regulates cell wall α-glucan biosynthesis through the protein kinase Pck2p. Mol. Biol. Cell.

[38] Barba G., Soto T., Madrid M., Nunez A., Vicente J., Gacto M., Cansado J. (2008). Activation of the cell integrity pathway is channelled through diverse signalling elements in fission yeast. Cell Signal.

[39] Cortés J.C.G., Sato M., Muñoz J., Moreno M.B., Clemente-Ramos J.A., Ramos M., Okada H., Osumi M., Durán A., Ribas J.C. (2012). Fission yeast Ags1 confers the essential septum strength needed for safe gradual cell abscission. J. Cell Biol..

[40] Nakano K., Imai J., Arai R., Toh E.A., Matsui Y., Mabuchi I. (2002). The small GTPase Rho3 and the diaphanous/formin For3 function in polarized cell growth in fission yeast. J. Cell Sci..

[41] Nakano K., Toya M., Yoneda A., Asami Y., Yamashita A., Kamasawa N., Osumi M., Yamamoto M. (2011). Pob1 ensures cylindrical cell shape by coupling two distinct Rho signaling events during secretory vesicle targeting. Traffic.

[42] Wang H., Tang X., Balasubramanian M.K. (2003). Rho3p regulates cell separation by modulating exocyst function in *Schizosaccharomyces pombe*. Genetics.

[43] Kita A., Li C., Yu Y., Umeda N., Doi A., Yasuda M., Ishiwata S., Taga A., Horiuchi Y., Sugiura R. (2011). Role of the Small GTPase Rho3 in Golgi/Endosome trafficking through functional interaction with adaptin in fission yeast. PLoS ONE.

[44] Yu Y., Li C., Kita A., Katayama Y., Kubouchi K., Udo M., Imanaka Y., Ueda S., Masuko T., Sugiura R. (2013). Sip1, an AP-1 accessory protein in fission yeast, is required for localization of Rho3 GTPase. PLoS ONE.

[45] Munoz S., Manjon E., Sanchez Y. (2014). The putative exchange factor Gef3p interacts with Rho3p GTPase and the septin ring during cytokinesis in fission yeast. J. Biol. Chem..

[46] Sedwick C. (2013). Palmitoylation: A new regulatory role in meiosis. PLoS Biol..

[47] Nakano K., Mutoh T., Arai R., Mabuchi I. (2003). The small GTPase Rho4 is involved in controlling cell morphology and septation in fission yeast. Genes Cells.

[48] Santos B., Gutierrez J., Calonge T.M., Perez P. (2003). Novel Rho GTPase involved in cytokinesis and cell wall integrity in the fission yeast *Schizosaccharomyces pombe*. Eukaryot. Cell.

[49] Santos B., Martin-Cuadrado A.B., Vazquez de Aldana C.R., del Rey F., Perez P. (2005). Rho4 GTPase is involved in secretion of glucanases during fission yeast cytokinesis. Eukaryot. Cell.

[50] Perez P., Portales E., Santos B. (2015). Rho4 interaction with exocyst and septins regulates cell separation in fission yeast. Microbiology.

[51] Doi A., Kita A., Kanda Y., Uno T., Asami K., Satoh R., Nakano K., Sugiura R. (2015). Geranylgeranyltransferase Cwg2-Rho4/Rho5 module is implicated in the Pmk1 MAP kinase-mediated cell wall integrity pathway in fission yeast. Genes Cells.

[52] Nakano K., Arai R., Mabuchi I. (2005). Small GTPase Rho5 is a functional homologue of Rho1, which controls cell shape and septation in fission yeast. FEBS Lett..

[53] Rincon S.A., Santos B., Perez P. (2006). Fission yeast Rho5p GTPase is a functional paralogue of Rho1p that plays a role in survival of spores and stationary-phase cells. Eukaryot. Cell.

[54] Johnson D.I. (1999). Cdc42: An essential Rho-type GTPase controlling eukaryotic cell polarity. Microbiol. Mol. Biol. Rev..

[55] Merla A., Johnson D.I. (2000). The Cdc42p GTPase is targeted to the site of cell division in the fission yeast *Schizosaccharomyces pombe*. Eur J. Cell Biol..

[56] Bendezu F.O., Vincenzetti V., Vavylonis D., Wyss R., Vogel H., Martin S.G. (2015). Spontaneous Cdc42 polarization independent of GDI-mediated extraction and actin-based trafficking. PLoS Biol..

[57] Chang E.C., Barr M., Wang Y., Jung V., Xu H.P., Wigler M.H. (1994). Cooperative interaction of *S. pombe* proteins required for mating and morphogenesis. Cell.

[58] Feierbach B., Chang F. (2001). Roles of the fission yeast formin For3p in cell polarity, actin cable formation and symmetric cell division. Curr. Biol..

[59] Coll P.M., Trillo Y., Ametzazurra A., Perez P. (2003). Gef1p, a new guanine nucleotide exchange factor for Cdc42p, regulates polarity in *Schizosaccharomyces pombe*. Mol. Biol. Cell.

[60] Martin S.G., Rincon S.A., Basu R., Perez P., Chang F. (2007). Regulation of the formin For3p by Cdc42p and Bud6p. Mol. Biol. Cell.

[61] Das M., Wiley D.J., Chen X., Shah K., Verde F. (2009). The conserved NDR kinase Orb6 controls polarized cell growth by spatial regulation of the small GTPase Cdc42. Curr. Biol..

[62] Rincon S.A., Ye Y., Villar-Tajadura M.A., Santos B., Martin S.G., Perez P. (2009). Pob1 participates in the Cdc42 regulation of fission yeast actin cytoskeleton. Mol. Biol. Cell.

[63] Perez P., Rincón S.A. (2010). Rho GTPases: Regulation of cell polarity and growth in yeasts. Biochem. J..

[64] Bendezu F.O., Martin S.G. (2011). Actin cables and the exocyst form two independent morphogenesis pathways in the fission yeast. Mol. Biol. Cell.

[65] Kelly F.D., Nurse P. (2011). Spatial control of Cdc42 activation determines cell width in fission yeast. Mol. Biol. Cell.

[66] Kelly F.D., Nurse P. (2011). *De novo* growth zone formation from fission yeast spheroplasts. PLoS ONE.

[67] Das M., Drake T., Wiley D.J., Buchwald P., Vavylonis D., Verde F. (2012). Oscillatory dynamics of Cdc42 GTPase in the control of polarized growth. Science.

[68] Martin S.G., Arkowitz R.A. (2014). Cell polarization in budding and fission yeasts. FEMS Microbiol. Rev..

[69] Rincon S.A., Estravis M., Perez P. (2014). Cdc42 regulates polarized growth and cell integrity in fission yeast. Biochem. Soc. Trans..

[70] Das M., Nunez I., Rodriguez M., Wiley D.J., Rodriguez J., Sarkeshik A., Yates J.R., Buchwald P., Verde F. (2015). Phosphorylation-dependent inhibition of Cdc42 GEF Gef1 by 14-3-3 protein Rad24 spatially regulates Cdc42 GTPase activity and oscillatory dynamics during cell morphogenesis. Mol. Biol. Cell.

[71] Martin S.G. (2015). Spontaneous cell polarization: Feedback control of Cdc42 GTPase breaks cellular symmetry. Bioessays.

[72] Chiou J.G., Balasubramanian M.K., Lew D.J. (2017). Cell Polarity in Yeast. Annu. Rev. Cell Dev. Biol..

[73] Gallo Castro D., Martin S.G. (2018). Differential GAP requirement for Cdc42-GTP polarization during proliferation and sexual reproduction. J. Cell Biol..

[74] Hercyk B.S., Rich-Robinson J., Mitoubsi A.S., Harrell M.A., Das M.E. (2019). A novel interplay between GEFs orchestrates Cdc42 activity during cell polarity and cytokinesis. J. Cell Sci..

[75] Hercyk B.S., Das M.E. (2019). F-BAR Cdc15 promotes Cdc42 activation during cytokinesis and cell polarization in *Schizosaccharomyces pombe*. Genetics.

[76] Lamas I., Merlini L., Vještica A., Vincenzetti V., Martin S.G. (2020). Optogenetics reveals Cdc42 local activation by scaffold-mediated positive feedback and Ras GTPase. PLoS Biol..

[77] Perez P., Soto T., Gomez-Gil E., Cansado J. (2019). Functional interaction between Cdc42 and the stress MAPK signaling pathway during the regulation of fission yeast polarized growth. Int. Microbiol..

[78] Merla A., Johnson D.I. (2001). The *Schizosaccharomyces pombe* Cdc42p GTPase signals through Pak2p and the Mkh1p-Pek1p-Spm1p MAP kinase pathway. Curr. Genet..

[79] Coll P.M., Rincon S.A., Izquierdo R.A., Perez P. (2007). Hob3p, the fission yeast ortholog of human BIN3, localizes Cdc42p to the division site and regulates cytokinesis. EMBO J..

[80] Rincon S., Coll P.M., Perez P. (2007). Spatial regulation of Cdc42 during cytokinesis. Cell Cycle.

[81] Loo T.H., Balasubramanian M. (2008). *Schizosaccharomyces pombe* Pak-related protein, Pak1p/Orb2p, phosphorylates myosin regulatory light chain to inhibit cytokinesis. J. Cell Biol..

[82] Lo Presti L., Chang F., Martin S.G. (2012). Myosin Vs organize actin cables in fission yeast. Mol. Biol. Cell.

[83] Coffman V.C., Sees J.A., Kovar D.R., Wu J.Q. (2013). The formins Cdc12 and For3 cooperate during contractile ring assembly in cytokinesis. J. Cell Biol..

[84] Gu Y., Oliferenko S. (2015). Comparative biology of cell division in the fission yeast clade. Curr. Opin. Microbiol..

[85] Wang N., Lee I.J., Rask G., Wu J.Q. (2016). Roles of the TRAPP-II complex and the exocyst in membrane deposition during fission yeast cytokinesis. PLoS Biol..

[86] Wei B., Hercyk B.S., Mattson N., Mohammadi A., Rich J., DeBruyne E., Clark M.M., Das M. (2016). Unique spatiotemporal activation pattern of Cdc42 by Gef1 and Scd1 promotes different events during cytokinesis. Mol. Biol. Cell.

[87] Pollard L.W., Bookwalter C.S., Tang Q., Krementsova E.B., Trybus K.M., Lowey S. (2017). Fission yeast myosin Myo2 is down-regulated in actin affinity by light chain phosphorylation. Proc. Natl. Acad. Sci. USA.

[88] Hercyk B.S., Onwubiko U.N., Das M.E. (2019). Coordinating septum formation and the actomyosin ring during cytokinesis in *Schizosaccharomyces pombe*. Mol. Microbiol..

[89] Magliozzi J.O., Sears J., Brady M., Opalko H.E., Kettenbach A.N., Moseley J.B. (2019). Defining how Pak1 regulates cell polarity and cell division in fission yeast. bioRxiv.

[90] Onwubiko U.N., Mlynarczyk P.J., Wei B., Habiyaremye J., Clack A., Abel S.M., Das M.E. (2019). A Cdc42 GEF, Gef1, through endocytosis organizes F-BAR Cdc15 along the actomyosin ring and promotes concentric furrowing. J. Cell Sci..

[91] Gómez-Gil E., Martín-García R., Vicente-Soler J., Franco A., Vázquez-Marín B., Prieto-Ruiz F., Soto T., Pérez P., Madrid M., Cansado J. (2020). Stress-activated MAPK signaling controls fission yeast actomyosin ring integrity by modulating formin For3 levels. eLife.

[92] Magliozzi J.O., Sears J., Cressey L., Brady M., Opalko H.E., Kettenbach A.N., Moseley J.B. (2020). Fission yeast Pak1 phosphorylates anillin-like Mid1 for spatial control of cytokinesis. J. Cell Biol..

[93] Bendezu F.O., Martin S.G. (2013). Cdc42 explores the cell periphery for mate selection in fission yeast. Curr. Biol..

[94] Merlini L., Khalili B., Bendezu F.O., Hurwitz D., Vincenzetti V., Vavylonis D., Martin S.G. (2016). Local pheromone release from dynamic polarity sites underlies cell-cell pairing during yeast mating. Curr. Biol..

[95] Merlini L., Khalili B., Dudin O., Michon L., Vincenzetti V., Martin S.G. (2018). Inhibition of Ras activity coordinates cell fusion with cell-cell contact during yeast mating. J. Cell Biol..

[96] Khalili B., Merlini L., Vincenzetti V., Martin S.G., Vavylonis D. (2018). Exploration and stabilization of Ras1 mating zone: A mechanism with positive and negative feedbacks. PLoS Comput. Biol..

[97] Wang H., Tang X., Liu J., Trautmann S., Balasundaram D., McCollum D., Balasubramanian M.K. (2002). The multiprotein exocyst complex is essential for cell separation in *Schizosaccharomyces pombe*. Mol. Biol. Cell.

[98] Nakano K., Arai R., Mabuchi I. (1997). The small GTP-binding protein Rho1 is a multifunctional protein that regulates actin localization, cell polarity, and septum formation in the fission yeast *Schizosaccharomyces pombe*. Genes Cells.

[99] Ozaki K., Tanaka K., Imamura H., Hihara T., Kameyama T., Nonaka H., Hirano H., Matsuura Y., Takai Y. (1996). Rom1p and Rom2p are GDP/GTP exchange proteins (GEPs) for the Rho1p small GTP binding protein in *Saccharomyces cerevisiae*. EMBO J..

[100] Schmidt A., Bickle M., Beck T., Hall M.N. (1997). The yeast phosphatidylinositol kinase homolog TOR2 activates RHO1 and RHO2 via the exchange factor ROM2. Cell.

[101] Ye Y., Lee I.J., Runge K.W., Wu J.Q. (2012). Roles of putative Rho-GEF Gef2 in division-site positioning and contractile-ring function in fission yeast cytokinesis. Mol. Biol. Cell.

[102] Zhu Y.H., Ye Y., Wu Z., Wu J.Q. (2013). Cooperation between Rho-GEF Gef2 and its binding partner Nod1 in the regulation of fission yeast cytokinesis. Mol. Biol. Cell.

[103] Nakano K., Mutoh T., Mabuchi I. (2001). Characterization of GTPase-activating proteins for the function of the Rho-family small GTPases in the fission yeast *Schizosaccharomyces pombe*. Genes Cells.

[104] Nakano K., Mabuchi I. (1995). Isolation and sequencing of two cDNA clones encoding Rho proteins from the fission yeast *Schizosaccharomyces pombe*. Gene.

[105] Soto T., Villar-Tajadura M.A., Madrid M., Vicente J., Gacto M., Perez P., Cansado J. (2010). Rga4 modulates the activity of the fission yeast cell integrity MAPK pathway by acting as a Rho2 GTPase-activating protein. J. Biol. Chem..

[106] Iwaki N., Karatsu K., Miyamoto M. (2003). Role of guanine nucleotide exchange factors for Rho family GTPases in the regulation of cell morphology and actin cytoskeleton in fission yeast. Biochem. Biophys. Res. Commun..

[107] Wang N., Wang M., Zhu Y.H., Grosel T.W., Sun D., Kudryashov D.S., Wu J.Q. (2015). The Rho-GEF Gef3 interacts with the septin complex and activates the GTPase Rho4 during fission yeast cytokinesis. Mol. Biol. Cell.

[108] Chen C.R., Li Y.C., Chen J., Hou M.C., Papadaki P., Chang E.C. (1999). Moe1, a conserved protein in *Schizosaccharomyces pombe*, interacts with a Ras effector, Scd1, to affect proper spindle formation. Proc. Natl. Acad. Sci. USA.

[109] Chang E., Bartholomeusz G., Pimental R., Chen J., Lai H., Wang L., Yang P., Marcus S. (1999). Direct binding and *in vivo* regulation of the fission yeast p21-activated kinase Shk1 by the SH3 domain protein Scd2. Mol. Cell. Biol..

[110] Endo M., Shirouzu M., Yokoyama S. (2003). The Cdc42 binding and scaffolding activities of the fission yeast adaptor protein Scd2. J. Biol. Chem..

[111] Murray J.M., Johnson D.I. (2001). The Cdc42p GTPase and its regulators Nrf1p and Scd1p are involved in endocytic trafficking in the fission yeast *Schizosaccharomyces pombe*. J. Biol. Chem..

[112] Li Y.C., Chen C.R., Chang E.C. (2000). Fission yeast Ras1 effector Scd1 interacts with the spindle and affects its proper formation. Genetics.

[113] Tay Y.D., Leda M., Goryachev A.B., Sawin K.E. (2018). Local and global Cdc42 guanine nucleotide exchange factors for fission yeast cell polarity are coordinated by microtubules and the Tea1-Tea4-Pom1 axis. J. Cell Sci..

[114] McDonald N.A., Lind A.L., Smith S.E., Li R., Gould K.L. (2017). Nanoscale architecture of the *Schizosaccharomyces pombe* contractile ring. eLife.

[115] Muñoz S., Manjón E., García P., Sunnerhagen P., Sánchez Y. (2014). The checkpoint-dependent nuclear accumulation of Rho1p exchange factor Rgf1p is important for tolerance to chronic replication stress. Mol. Biol. Cell.

[116] Manjón E., Edreira T., Muñoz S., Sánchez Y. (2017). Rgf1p (Rho1p GEF) is required for double-strand break repair in fission yeast. Nucleic Acids Res..

[117] Garcia P., Garcia I., Marcos F., de Garibay G.R., Sanchez Y. (2009). Fission yeast Rgf2p is a Rho1p guanine nucleotide exchange factor required for spore wall maturation and for the maintenance of cell integrity in the absence of Rgf1p. Genetics.

[118] Ren L., Willet A.H., Roberts-Galbraith R.H., McDonald N.A., Feoktistova A., Chen J.S., Huang H., Guillen R., Boone C., Sidhu S.S. (2015). The Cdc15 and Imp2 SH3 domains cooperatively scaffold a network of proteins that redundantly ensure efficient cell division in fission yeast. Mol. Biol. Cell.

[119] Davidson R., Laporte D., Wu J.-Q. (2014). Regulation of Rho-GEF Rgf3 by the arrestin Art1 in fission yeast cytokinesis. Mol. Biol. Cell.

[120] Rustici G., Mata J., Kivinen K., Lió P., Penkett C.J., Burns G., Hayles J., Brazma A., Nurse P., Bähler J. (2004). Periodic gene expression program of the fission yeast cell cycle. Nat. Genet..

[121] Das M., Wiley D.J., Medina S., Vincent H.A., Larrea M., Oriolo A., Verde F. (2007). Regulation of cell diameter, For3p localization, and cell symmetry by fission yeast Rho-GAP Rga4p. Mol. Biol. Cell.

[122] Tatebe H., Nakano K., Maximo R., Shiozaki K. (2008). Pom1 DYRK regulates localization of the Rga4 GAP to ensure bipolar activation of Cdc42 in fission yeast. Curr. Biol..

[123] Kokkoris K., Gallo Castro D., Martin S.G. (2014). The Tea4-PP1 landmark promotes local growth by dual Cdc42 GEF recruitment and GAP exclusion. J. Cell Sci..

[124] Calonge T.M., Arellano M., Coll P.M., Perez P. (2003). Rga5p is a specific Rho1p GTPase-activating protein that regulates cell integrity in *Schizosaccharomyces pombe*. Mol. Microbiol..

[125] Revilla-Guarinos M.T., Martin-Garcia R., Villar-Tajadura M.A., Estravis M., Coll P.M., Perez P. (2016). Rga6 is a fission yeast Rho GAP involved in Cdc42 regulation of polarized growth. Mol. Biol. Cell.

[126] Martín-García R., Coll P.M., Pérez P. (2014). F-BAR domain protein Rga7 collaborates with Cdc15 and Imp2 to ensure proper cytokinesis in fission yeast. J. Cell Sci..

[127] Arasada R., Pollard T.D. (2015). A role for F-BAR protein Rga7p during cytokinesis in *S. pombe*. J. Cell Sci..

[128] Yang P., Qyang Y., Bartholomeusz G., Zhou X., Marcus S. (2003). The novel Rho GTPase-activating protein family protein, Rga8, provides a potential link between Cdc42/p21-activated kinase and Rho signaling pathways in the fission yeast, *Schizosaccharomyces pombe*. J. Biol. Chem..

[129] Murray J.M., Johnson D.I. (2000). Isolation and characterization of Nrf1p, a novel negative regulator of the Cdc42p GTPase in *Schizosaccharomyces pombe*. Genetics.

[130] Ottilie S., Miller P.J., Johnson D.I., Creasy C.L., Sells M.A., Bagrodia S., Forsburg S.L., Chernoff J. (1995). Fission yeast *pak1^+^* encodes a protein kinase that interacts with Cdc42p and is involved in the control of cell polarity and mating. EMBO J..

[131] Fukui Y., Yamamoto M. (1988). Isolation and characterization of *Schizosaccharomyces pombe* mutants phenotypically similar to ras1. Mol. Gen. Genet..

[132] Hirota K., Tanaka K., Ohta K., Yamamoto M. (2003). Gef1p and Scd1p, the Two GDP-GTP exchange factors for Cdc42p, form a ring structure that shrinks during cytokinesis in *Schizosaccharomyces pombe*. Mol. Biol. Cell.

[133] Davì V., Tanimoto H., Ershov D., Haupt A., De Belly H., Le Borgne R., Couturier E., Boudaoud A., Minc N. (2018). Mechanosensation dynamically coordinates polar growth and cell wall assembly to promote cell survival. Dev. Cell.

[134] Bohnert K.A., Gould K.L. (2012). Cytokinesis-Based constraints on polarized cell growth in fission yeast. PLoS Genet..

[135] Makushok T., Alves P., Huisman S.M., Kijowski A.R., Brunner D. (2016). Sterol-Rich membrane domains define fission yeast cell polarity. Cell.

[136] Estravis M., Rincon S.A., Santos B., Perez P. (2011). Cdc42 regulates multiple membrane traffic events in fission yeast. Traffic.

[137] Estravis M., Rincon S.A., Portales E., Perez P., Santos B. (2017). Cdc42 activation state affects its localization and protein levels in fission yeast. Microbiology.

[138] Sohrmann M., Peter M. (2003). Polarizing without a c(l)ue. Trends Cell Biol..

[139] Wedlich-Soldner R., Altschuler S., Wu L., Li R. (2003). Spontaneous cell polarization through actomyosin-based delivery of the Cdc42 GTPase. Science.

[140] Witte K., Strickland D., Glotzer M. (2017). Cell cycle entry triggers a switch between two modes of Cdc42 activation during yeast polarization. eLife.

[141] Woods B., Kuo C.C., Wu C.F., Zyla T.R., Lew D.J. (2015). Polarity establishment requires localized activation of Cdc42. J. Cell Biol..

[142] Rapali P., Mitteau R., Braun C., Massoni-Laporte A., Ünlü C., Bataille L., Arramon F.S., Gygi S.P., McCusker D. (2017). Scaffold-mediated gating of Cdc42 signalling flux. eLife.

[143] Kuo C.C., Savage N.S., Chen H., Wu C.F., Zyla T.R., Lew D.J. (2014). Inhibitory GEF phosphorylation provides negative feedback in the yeast polarity circuit. Curr. Biol..

[144] Okada S., Leda M., Hanna J., Savage N.S., Bi E., Goryachev A.B. (2013). Daughter cell identity emerges from the Interplay of Cdc42, Septins, and Exocytosis. Dev. Cell.

[145] Lambert J.M., Lambert Q.T., Reuther G.W., Malliri A., Siderovski D.P., Sondek J., Collard J.G., Der C.J. (2002). Tiam1 mediates Ras activation of Rac by a PI(3)K-independent mechanism. Nat. Cell Biol..

[146] Bendezu F.O., Vincenzetti V., Martin S.G. (2012). Fission yeast Sec3 and Exo70 are transported on actin cables and localize the exocyst complex to cell poles. PLoS ONE.

[147] Dodgson J., Chessel A., Vaggi F., Giordan M., Yamamoto M., Arai K., Madrid M., Geymonat M., Abenza J.F., Cansado J. (2017). Reconstructing regulatory pathways by systematically mapping protein localization interdependency networks. bioRxiv.

[148] Gulli M.P., Jaquenoud M., Shimada Y., Niederhäuser G., Wiget P., Peter M. (2000). Phosphorylation of the Cdc42 exchange factor Cdc24 by the PAK-like kinase Cla4 may regulate polarized growth in yeast. Mol. Cell.

[149] Martin S.G., McDonald W.H., Yates J.R., Chang F. (2005). Tea4p links microtubule plus ends with the formin For3p in the establishment of cell polarity. Dev. Cell.

[150] Tatebe H., Shimada K., Uzawa S., Morigasaki S., Shiozaki K. (2005). Wsh3/Tea4 is a novel cell-end factor essential for bipolar distribution of Tea1 and protects cell polarity under environmental stress in *S. pombe*. Curr. Biol..

[151] Martin S.G., Berthelot-Grosjean M. (2009). Polar gradients of the DYRK-family kinase Pom1 couple cell length with the cell cycle. Nature.

[152] Alvarez-Tabares I., Grallert A., Ortiz J.M., Hagan I.M. (2007). *Schizosaccharomyces pombe* Protein Phosphatase 1 in mitosis, endocytosis and a partnership with Wsh3/Tea4 to control polarised growth. J. Cell Sci..

[153] Hachet O., Berthelot-Grosjean M., Kokkoris K., Vincenzetti V., Moosbrugger J., Martin S.G. (2011). A phosphorylation cycle shapes gradients of the DYRK family kinase Pom1 at the plasma membrane. Cell.

[154] Bi E., Park H.-O. (2012). Cell polarization and cytokinesis in budding yeast. Genetics.

[155] Mata J., Nurse P. (1997). tea1 and the microtubular cytoskeleton are important for generating global spatial order within the fission yeast cell. Cell.

[156] Bahler J., Pringle J.R. (1998). Pom1p, a fission yeast protein kinase that provides positional information for both polarized growth and cytokinesis. Genes Dev..

[157] Rich-Robinson J., Russell A., Mancini E., Das M. (2020). Cdc42 reactivation at growth sites is regulated by cell-cycle-dependent removal of its GAP Rga4 in fission yeast. bioRxiv.

[158] Zheng S., Zheng B., Liu Z., Wei W., Fu C. (2021). The Cdc42 GTPase activating protein Rga6 promotes the cortical localization of Septin. bioRxiv.

[159] Iwase M., Luo J., Nagaraj S., Longtine M., Kim H.B., Haarer B.K., Caruso C., Tong Z., Pringle J.R., Bi E. (2006). Role of a Cdc42p effector pathway in recruitment of the yeast septins to the presumptive bud site. Mol. Biol. Cell.

[160] Pino M.R., Nuñez I., Chen C., Das M.E., Wiley D.J., D’Urso G., Buchwald P., Vavylonis D., Verde F. (2020). Cdc42 GTPase activating proteins (GAPs) maintain generational inheritance of cell polarity and cell shape in fission yeast. bioRxiv.

[161] Bugaj L.J., Choksi A.T., Mesuda C.K., Kane R.S., Schaffer D.V. (2013). Optogenetic protein clustering and signaling activation in mammalian cells. Nat. Methods.

[162] Liu H., Yu X., Li K., Klejnot J., Yang H., Lisiero D., Lin C. (2008). Photoexcited CRY2 interacts with CIB1 to regulate transcription and floral initiation in *Arabidopsis*. Science.

[163] Lamas I., Weber N., Martin S.G. (2020). Activation of Cdc42 GTPase upon CRY2-induced cortical recruitment is antagonized by GAPs in fission yeast. Cells.

[164] Sells M.A., Barratt J.T., Caviston J., Ottilie S., Leberer E., Chernoff J. (1998). Characterization of Pak2p, a pleckstrin homology domain-containing, p21-activated protein kinase from fission yeast. J. Biol. Chem..

[165] Marcus S., Polverino A., Chang E., Robbins D., Cobb M.H., Wigler M.H. (1995). Shk1, a homolog of the *Saccharomyces cerevisiae* Ste20 and mammalian p65PAK protein kinases, is a component of a Ras/Cdc42 signaling module in the fission yeast *Schizosaccharomyces pombe*. Proc. Natl. Acad. Sci. USA.

[166] Verde F., Wiley D.J., Nurse P. (1998). Fission yeast Orb6, a Ser/Thr protein kinase related to mammalian Rho kinase and myotonic dystrophy kinase, is required for maintenance of cell polarity and coordinates cell morphogenesis with the cell cycle. Proc. Natl. Acad. Sci. USA.

[167] Yang P., Kansra S., Pimental R.A., Gilbreth M., Marcus S. (1998). Cloning and characterization of *shk2,* a gene encoding a novel p21-activated protein kinase from fission yeast. J. Biol. Chem..

[168] Qyang Y., Yang P., Du H., Lai H., Kim H., Marcus S. (2002). The p21-activated kinase, Shk1, is required for proper regulation of microtubule dynamics in the fission yeast *Schizosaccharomyces pombe*. Mol. Microbiol..

[169] Tu H., Wigler M. (1999). Genetic evidence for Pak1 autoinhibition and its release by Cdc42. Mol. Cell. Biol..

[170] Lei M., Lu W., Meng W., Parrini M.C., Eck M.J., Mayer B.J., Harrison S.C. (2000). Structure of PAK1 in an autoinhibited conformation reveals a multistage activation switch. Cell.

[171] Morreale A., Venkatesan M., Mott H.R., Owen D., Nietlispach D., Lowe P.N., Laue E.D. (2000). Structure of Cdc42 bound to the GTPase binding domain of PAK. Nat. Struct. Biol..

[172] Kim H., Yang P., Catanuto P., Verde F., Lai H., Du H., Chang F., Marcus S. (2003). The kelch repeat protein, Tea1, is a potential substrate target of the p21-activated kinase, Shk1, in the fission yeast *Schizosaccharomyces pombe*. J. Biol. Chem..

[173] Geymonat M., Chessel A., Dodgson J., Punter H., Horns F., Nagy A.C., Salas R.E.C. (2018). Activation of polarized cell growth by inhibition of cell polarity. bioRxiv.

[174] Martin S.G., Chang F. (2006). Dynamics of the formin For3p in actin cable assembly. Curr. Biol..

[175] Scott B.J., Neidt E.M., Kovar D.R. (2011). The functionally distinct fission yeast formins have specific actin-assembly properties. Mol. Biol. Cell.

[176] Motegi F., Arai R., Mabuchi I. (2001). Identification of two type V myosins in fission yeast, one of which functions in polarized cell growth and moves rapidly in the cell. Mol. Biol. Cell.

[177] Cortes J.C., Carnero E., Ishiguro J., Sanchez Y., Duran A., Ribas J.C. (2005). The novel fission yeast (1,3)β-D-glucan synthase catalytic subunit Bgs4p is essential during both cytokinesis and polarized growth. J. Cell Sci..

[178] Mulvihill D.P., Edwards S.R., Hyams J.S. (2006). A critical role for the type V myosin, Myo52, in septum deposition and cell fission during cytokinesis in *Schizosaccharomyces pombe*. Cell. Motil. Cytoskelet..

[179] Glynn J.M., Lustig R.J., Berlin A., Chang F. (2001). Role of Bud6p and Tea1p in the interaction between actin and microtubules for the establishment of cell polarity in fission yeast. Curr. Biol..

[180] Dong Y., Pruyne D., Bretscher A. (2003). Formin-dependent actin assembly is regulated by distinct modes of Rho signaling in yeast. J. Cell Biol..

[181] TerBush D.R., Maurice T., Roth D., Novick P. (1996). The Exocyst is a multiprotein complex required for exocytosis in *Saccharomyces cerevisiae*. EMBO J..

[182] He B., Guo W. (2009). The exocyst complex in polarized exocytosis. Curr. Opin. Cell Biol..

[183] Finger F.P., Hughes T.E., Novick P. (1998). Sec3p is a spatial landmark for polarized secretion in budding yeast. Cell.

[184] Mutavchiev D.R., Leda M., Sawin K.E. (2016). Remodeling of the fission yeast Cdc42 cell-polarity module via the Sty1 p38 Stress-Activated Protein Kinase pathway. Curr. Biol..

[185] Haupt A., Ershov D., Minc N. (2018). A positive feedback between growth and polarity provides directional persistency and flexibility to the process of tip growth. Curr. Biol..

[186] Brewster J.L., Gustin M.C. (2014). Hog1: 20 years of discovery and impact. Sci. Signal..

[187] Koyano T., Kume K., Konishi M., Toda T., Hirata D. (2010). Search for kinases related to transition of growth polarity in fission yeast. Biosci. Biotechnol. Biochem..

[188] Papp L., Sipiczki M., Holb I.J., Miklós I. (2014). Optimal conditions for mycelial growth of *Schizosaccharomyces japonicus* cells in liquid medium: It enables the molecular investigation of dimorphism. Yeast.

[189] Furuya K., Niki H. (2010). The DNA damage checkpoint regulates a transition between yeast and hyphal growth in *Schizosaccharomyces japonicus*. Mol. Cell. Biol..

[190] Furuya K., Niki H. (2012). Hyphal differentiation induced via a DNA damage checkpoint-dependent pathway engaged in crosstalk with nutrient stress signaling in *Schizosaccharomyces japonicus*. Curr. Genet..

[191] Chen H., Zhou X., Ren B., Cheng L. (2020). The regulation of hyphae growth in *Candida albicans*. Virulence.

[192] Gómez-Gil E., Franco A., Madrid M., Vázquez-Marín B., Gacto M., Fernández-Breis J., Vicente-Soler J., Soto T., Cansado J. (2019). Quorum sensing and stress-activated MAPK signaling repress yeast to hypha transition in the fission yeast *Schizosaccharomyces japonicus*. PLoS Genet..

[193] Mazur P., Baginsky W. (1996). In vitro activity of 1,3-β-D-glucan synthase requires the GTP-binding protein Rho1. J. Biol. Chem..

[194] Mukai H. (2003). The structure and function of PKN, a protein kinase having a catalytic domain homologous to that of PKC. J. Biochem..

[195] Estravis M., Rincon S., Perez P. (2012). Cdc42 regulation of polarized traffic in fission yeast. Commun. Integr. Biol..

[196] Deng L., Sugiura R., Ohta K., Tada K., Suzuki M., Hirata M., Nakamura S.-I., Shuntoh H., Kuno T. (2005). Phosphatidylinositol-4-phosphate 5-kinase regulates fission yeast cell integrity through a phospholipase c-mediated protein kinase C-independent pathway. J. Biol. Chem..

[197] Martín V., García B., Carnero E., Durán A., Sánchez Y. (2003). Bgs3p, a putative 1,3-β-glucan synthase subunit, is required for cell wall assembly in *Schizosaccharomyces pombe*. Eukaryot. Cell.

[198] Cruz S., Munoz S., Manjon E., Garcia P., Sanchez Y. (2013). The fission yeast cell wall stress sensor-like proteins Mtl2 and Wsc1 act by turning on the GTPase Rho1p but act independently of the cell wall integrity pathway. Microbiologyopen.

[199] Neeli-Venkata R., Celador R., Sanchez Y., Minc N. (2020). Detection of surface forces by a cell wall mechanosensor. bioRxiv.

[200] Davi V., Minc N. (2015). Mechanics and morphogenesis of fission yeast cells. Curr. Opin. Microbiol..

[201] Levin D.E. (2011). Regulation of cell wall biogenesis in *Saccharomyces cerevisiae*: The cell wall integrity signaling pathway. Genetics.

[202] Perez P., Cansado J. (2010). Cell integrity signaling and response to stress in fission yeast. Curr. Protein Pept. Sci..

[203] Guo S., Shen X., Yan G., Ma D., Bai X., Li S., Jiang Y. (2009). A Map kinase dependent feedback mechanism controls Rho1 GTPase and actin distribution in yeast. PLoS ONE.

[204] Kabeche R., Madrid M., Cansado J., Moseley J.B. (2015). Eisosomes regulate phosphatidylinositol 4,5-Bisphosphate (PI(4,5)P2) Cortical Clusters and Mitogen-activated Protein (MAP) kinase signaling upon osmotic stress. J. Biol. Chem..

[205] Franco A., Soto T., Martín-García R., Madrid M., Vázquez-Marín B., Vicente-Soler J., Coll P.M., Gacto M., Pérez P., Cansado J. (2017). Distinct functional relevance of dynamic GTPase cysteine methylation in fission yeast. Sci. Rep..

[206] Wang M., Casey P.J. (2016). Protein prenylation: Unique fats make their mark on biology. Nat. Rev. Mol. Cell. Biol..

[207] Cansado J. (2018). To finish things well: Cysteine methylation ensures selective GTPase membrane localization and signalling. Curr. Genet..

[208] Viana R.A., Pinar M., Soto T., Coll P.M., Cansado J., Perez P. (2013). Negative functional interaction between cell integrity MAPK pathway and Rho1 GTPase in fission yeast. Genetics.

[209] Kampmeyer C., Johansen J.V., Holmberg C., Karlson M., Gersing S.K., Bordallo H.N., Kragelund B.B., Lerche M.H., Jourdain I., Winther J.R. (2020). Mutations in a single signaling pathway allow cell growth in heavy water. ACS Synth. Biol..

[210] Madrid M., Soto T., Khong H.K., Franco A., Vicente J., Perez P., Gacto M., Cansado J. (2006). Stress-induced response, localization, and regulation of the Pmk1 cell integrity pathway in *Schizosaccharomyces pombe*. J. Biol. Chem..

[211] Kono K., Saeki Y., Yoshida S., Tanaka K., Pellman D. (2012). Proteasomal degradation resolves competition between cell polarization and cellular wound healing. Cell.

[212] Rincon S.A., Paoletti A. (2016). Molecular control of fission yeast cytokinesis. Semin. Cell Dev. Biol..

[213] Pollard T.D., Wu J.Q. (2010). Understanding cytokinesis: Lessons from fission yeast. Nat. Rev. Mol. Cell. Biol..

[214] Garcia Cortes J.C., Ramos M., Osumi M., Perez P., Ribas J.C. (2016). The Cell biology of fission yeast septation. Microbiol. Mol. Biol. Rev..

[215] Onwubiko U.N., Rich-Robinson J., Mustaf R.A., Das M.E. (2020). Cdc42 promotes Bgs1 recruitment for septum synthesis and glucanase localization for cell separation during cytokinesis in fission yeast. Small GTPases.

[216] Meitinger F., Boehm M.E., Hofmann A., Hub B., Zentgraf H., Lehmann W.D., Pereira G. (2011). Phosphorylation-dependent regulation of the F-BAR protein Hof1 during cytokinesis. Genes Dev..

[217] Bose I., Irazoqui J.E., Moskow J.J., Bardes E.S., Zyla T.R., Lew D.J. (2001). Assembly of scaffold-mediated complexes containing Cdc42p, the exchange factor Cdc24p, and the effector Cla4p required for cell cycle-regulated phosphorylation of Cdc24p. J. Biol. Chem..

[218] Versele M., Thorner J. (2004). Septin collar formation in budding yeast requires GTP binding and direct phosphorylation by the PAK, Cla4. J. Cell Biol..

[219] Muñoz J., Cortés J.C.G., Sipiczki M., Ramos M., Clemente-Ramos J.A., Moreno M.B., Martins I.M., Pérez P., Ribas J.C. (2013). Extracellular cell wall β(1,3)glucan is required to couple septation to actomyosin ring contraction. J. Cell Biol..

[220] Arasada R., Pollard T.D. (2014). Contractile ring stability in *S. pombe* depends on F-BAR protein Cdc15p and Bgs1p transport from the Golgi Complex. Cell Rep..

[221] Watanabe S., Okawa K., Miki T., Sakamoto S., Morinaga T., Segawa K., Arakawa T., Kinoshita M., Ishizaki T., Narumiya S. (2010). Rho and anillin-dependent control of mDia2 localization and function in cytokinesis. Mol. Biol. Cell.

[222] Yoshida S., Kono K., Lowery D.M., Bartolini S., Yaffe M.B., Ohya Y., Pellman D. (2006). Polo-Like Kinase Cdc5 controls the local activation of Rho1 to promote cytokinesis. Science.

[223] Cortés J.C., Pujol N., Sato M., Pinar M., Ramos M., Moreno B., Osumi M., Ribas J.C., Pérez P. (2015). Cooperation between Paxillin-like Protein Pxl1 and glucan synthase Bgs1 is essential for actomyosin ring stability and septum formation in fission yeast. PLoS Genet.

[224] Zhu Y.H., Hyun J., Pan Y.Z., Hopper J.E., Rizo J., Wu J.Q. (2018). Roles of the fission yeast UNC-13/Munc13 protein Ync13 in late stages of cytokinesis. Mol. Biol. Cell.

[225] Dundon S.E.R., Pollard T.D. (2020). Microtubule nucleation promoters Mto1 and Mto2 regulate cytokinesis in fission yeast. Mol. Biol. Cell.

[226] Madrid M., Núñez A., Soto T., Vicente-Soler J., Gacto M., Cansado J. (2007). Stress-activated protein kinase-mediated down-regulation of the cell integrity pathway mitogen-activated protein kinase Pmk1p by protein phosphatases. Mol. Biol. Cell.

[227] Satoh R., Morita T., Takada H., Kita A., Ishiwata S., Doi A., Hagihara K., Taga A., Matsumura Y., Tohda H. (2009). Role of the RNA-binding protein Nrd1 and Pmk1 mitogen-activated protein kinase in the regulation of myosin mRNA stability in fission yeast. Mol. Biol. Cell.

[228] Martín-García R., Arribas V., Coll P.M., Pinar M., Viana R.A., Rincón S.A., Correa-Bordes J., Ribas J.C., Pérez P. (2018). Paxillin-mediated recruitment of calcineurin to the contractile ring is required for the correct progression of cytokinesis in fission yeast. Cell Rep..

[229] Simanis V. (2015). Pombe’s thirteen—Control of fission yeast cell division by the septation initiation network. J. Cell Sci..

[230] Martin-Garcia R., Santos B. (2016). The price of independence: Cell separation in fission yeast. World J. Microbiol. Biotechnol..

[231] Gu Y., Yam C., Oliferenko S. (2015). Rewiring of cellular division site selection in evolution of fission yeasts. Curr. Biol..

[232] Gu Y., Oliferenko S. (2019). Cellular geometry scaling ensures robust division site positioning. Nat. Commun..

[233] Yonetani A., Lustig R.J., Moseley J.B., Takeda T., Goode B.L., Chang F. (2008). Regulation and targeting of the fission yeast formin cdc12p in cytokinesis. Mol. Biol. Cell.

[234] Park H.O., Bi E. (2007). Central roles of small GTPases in the development of cell polarity in yeast and beyond. Microbiol. Mol. Biol. Rev..

[235] Imai Y., Yamamoto M. (1994). The fission yeast mating pheromone P-factor: Its molecular structure, gene structure, and ability to induce gene expression and G1 arrest in the mating partner. Genes Dev..

[236] Davey J. (1992). Mating pheromones of the fission yeast *Schizosaccharomyces pombe*: Purification and structural characterization of M-factor and isolation and analysis of two genes encoding the pheromone. EMBO J..

[237] Tanaka K., Davey J., Imai Y., Yamamoto M. (1993). *Schizosaccharomyces pombe map3^+^* encodes the putative M-factor receptor. Mol. Cell. Biol..

[238] Kitamura K., Shimoda C. (1991). The *Schizosaccharomyces pombe mam2* gene encodes a putative pheromone receptor which has a significant homology with the *Saccharomyces cerevisiae* Ste2 protein. EMBO J..

[239] Nadin-Davis S.A., Nasim A. (1990). *Schizosaccharomyces pombe ras1* and *byr1* are functionally related genes of the ste family that affect starvation-induced transcription of mating-type genes. Mol. Cell. Biol..

[240] Wang Y., Xu H.P., Riggs M., Rodgers L., Wigler M. (1991). *byr2*, a *Schizosaccharomyces pombe* gene encoding a protein kinase capable of partial suppression of the Ras1 mutant phenotype. Mol. Cell. Biol..

[241] Hughes D.A., Ashworth A., Marshall C.J. (1993). Complementation of *byr1* in fission yeast by mammalian MAP kinase kinase requires coexpression of Raf kinase. Nature.

[242] Bar E.E., Ellicott A.T., Stone D.E. (2003). Gβγ recruits Rho1 to the site of polarized growth during mating in budding yeast. J. Biol. Chem..

[243] Hughes D.A., Yabana N., Yamamoto M. (1994). Transcriptional regulation of a Ras nucleotide-exchange factor gene by extracellular signals in fission yeast. J. Cell Sci..

[244] Nadin-Davis S.A., Nasim A., Beach D. (1986). Involvement of ras in sexual differentiation but not in growth control in fission yeast. EMBO J..

[245] Davey J. (1998). Fusion of a fission yeast. Yeast.

[246] Merlini L., Dudin O., Martin S.G. (2013). Mate and fuse: How yeast cells do it. Open Biol..

[247] Tu H., Barr M., Dong D.L., Wigler M. (1997). Multiple regulatory domains on the Byr2 protein kinase. Mol. Cell. Biol..

[248] Leeuw T., Fourest-Lieuvin A., Wu C., Chenevert J., Clark K., Whiteway M., Thomas D.Y., Leberer E. (1995). Pheromone response in yeast: Association of Bem1p with proteins of the MAP kinase cascade and actin. Science.

[249] Mata J., Bahler J. (2006). Global roles of Ste11p, cell type, and pheromone in the control of gene expression during early sexual differentiation in fission yeast. Proc. Natl. Acad. Sci. USA.

[250] Vještica A., Merlini L., Nkosi P.J., Martin S.G. (2018). Gamete fusion triggers bipartite transcription factor assembly to block re-fertilization. Nature.

[251] Zhang M.M., Wu P.Y., Kelly F.D., Nurse P., Hang H.C. (2013). Quantitative control of protein S-palmitoylation regulates meiotic entry in fission yeast. PLoS Biol..

[252] Yoo B.Y., Calleja G.B., Johnson B.F. (1973). Ultrastructural changes of the fission yeast (*Schizosaccharomyces pombe*) during ascospore formation. Arch. Mikrobiol..

[253] Garcia P., Tajadura V., Garcia I., Sanchez Y. (2006). Role of Rho GTPases and Rho-GEFs in the regulation of cell shape and integrity in fission yeast. Yeast.

[254] Hatanaka M., Shimoda C. (2001). The cyclic AMP/PKA signal pathway is required for initiation of spore germination in *Schizosaccharomyces pombe*. Yeast.

[255] Bonazzi D., Julien J.D., Romao M., Seddiki R., Piel M., Boudaoud A., Minc N. (2014). Symmetry breaking in spore germination relies on an interplay between polar cap stability and spore wall mechanics. Dev. Cell.

[256] Ellerbroek S.M., Wennerberg K., Burridge K. (2003). Serine phosphorylation negatively regulates RhoA in vivo. J. Biol. Chem..

[257] Forget M.A., Desrosiers R.R., Gingras D., Béliveau R. (2002). Phosphorylation states of Cdc42 and RhoA regulate their interactions with Rho GDP dissociation inhibitor and their extraction from biological membranes. Biochem. J..

[258] Tay Y.D., Leda M., Spanos C., Rappsilber J., Goryachev A.B., Sawin K.E. (2019). Fission yeast NDR/LATS kinase Orb6 regulates exocytosis via phosphorylation of the exocyst complex. Cell Rep..

